# Supplementary material for: Single-cell profiling reveals a memory B cell-like subtype of follicular lymphoma with increased transformation risk
Source: Nat Commun. 2022 Nov 9;13:6772. doi: 10.1038/s41467-022-34408-0 (PMC9646774; doi:10.1038/s41467-022-34408-0)
Supplement: Supplementary file 1 — Supplementary Information [file 41467_2022_34408_MOESM1_ESM.pdf]

SUPPLEMENTARY INFORMATION

for

“Single-cell profiling reveals a memory B cell-like subtype of follicular lymphoma with increased transformation risk”

Including

Supplementary Figures 1-25

Supplementary Tables 1-2

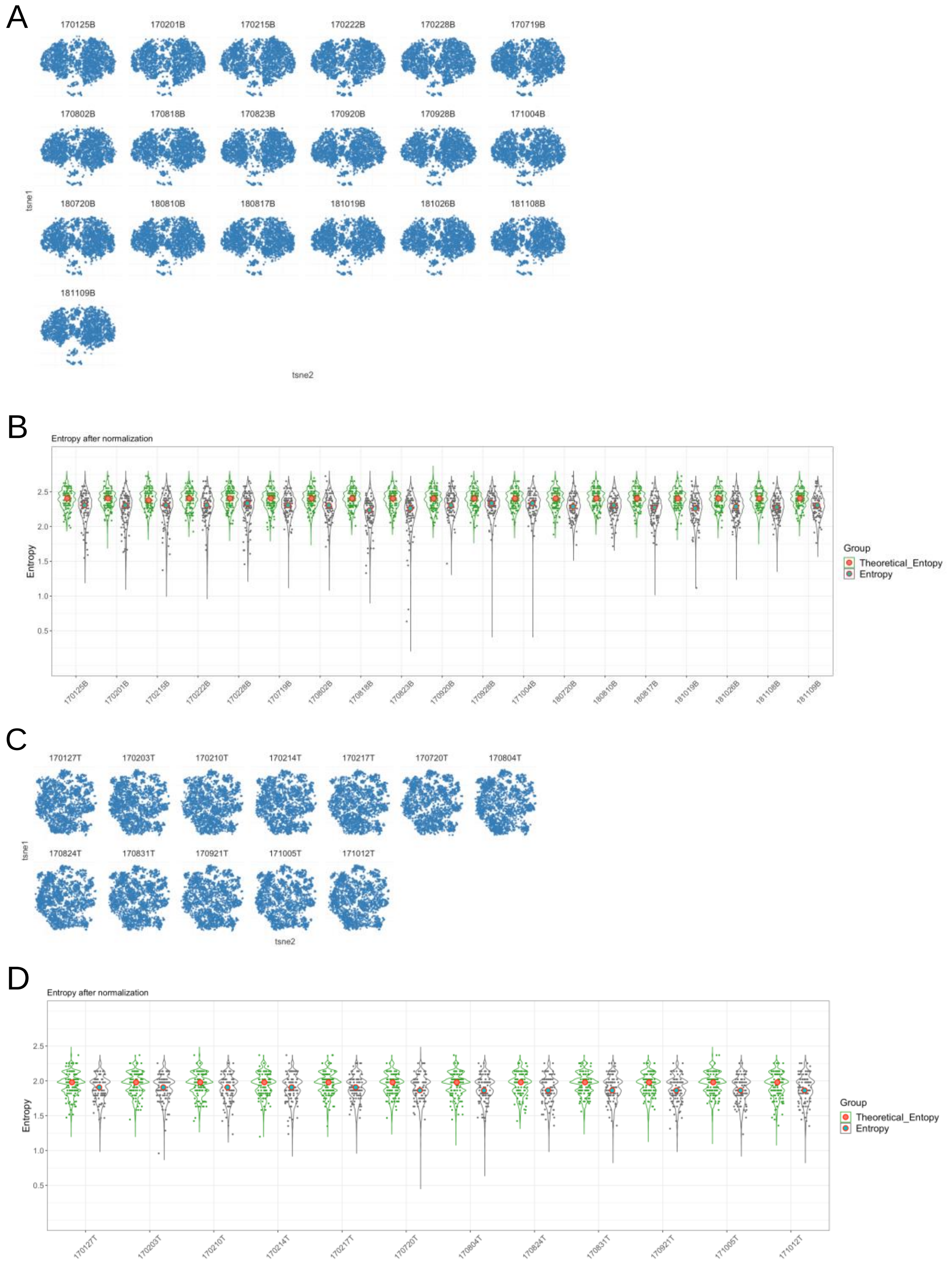

Supplementary Figure 1. CyTOF data is highly reproducible across acquisition batches

A,C) Dimensional reduction tSNE plots of **A**) B-cell and **C**) T-cell data from spiked-in, pooled reactive LN (rLN) controls collected across 19 and 12 acquisition batches, respectively. Each dot represents a single cell.

B,D) Entropy distribution plots for each acquisition run, as compared to theoretical maximum for **B**) B-cell and **D**) T-cell data. Each dot represents a single cell.

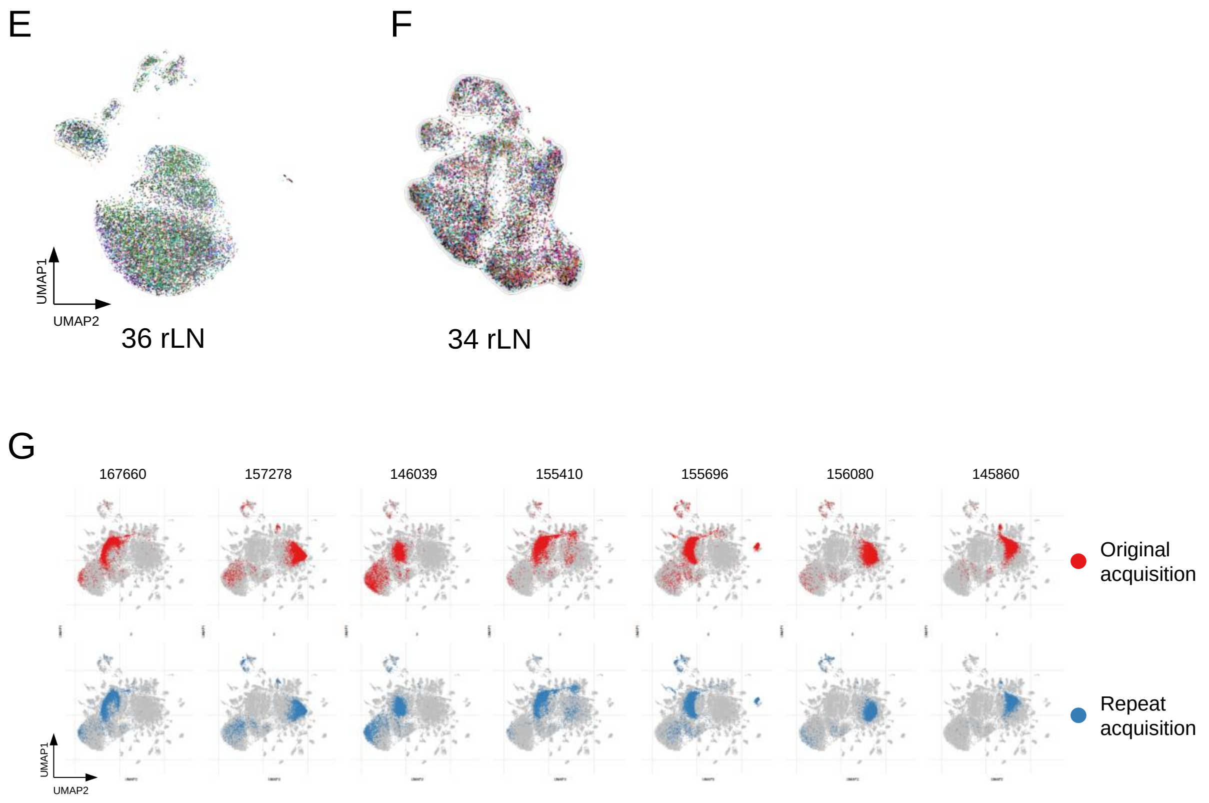

Supplementary Figure 1. CyTOF data is highly reproducible across acquisition batches (continued)

E,F) Dimensional reduction UMAP plots of **E**) B-cell and **F**) T-cell data from 36 and 34 rLN samples, respectively. Each dot represents a single cell. Dots are colored by patient sample.
G) UMAP plots of B-cell data as in **Fig 1A** from 7 FL samples stained and acquired on two separate occasions. Cells from the other 148 FL + 36 rLN samples in the global analysis colored in grey.

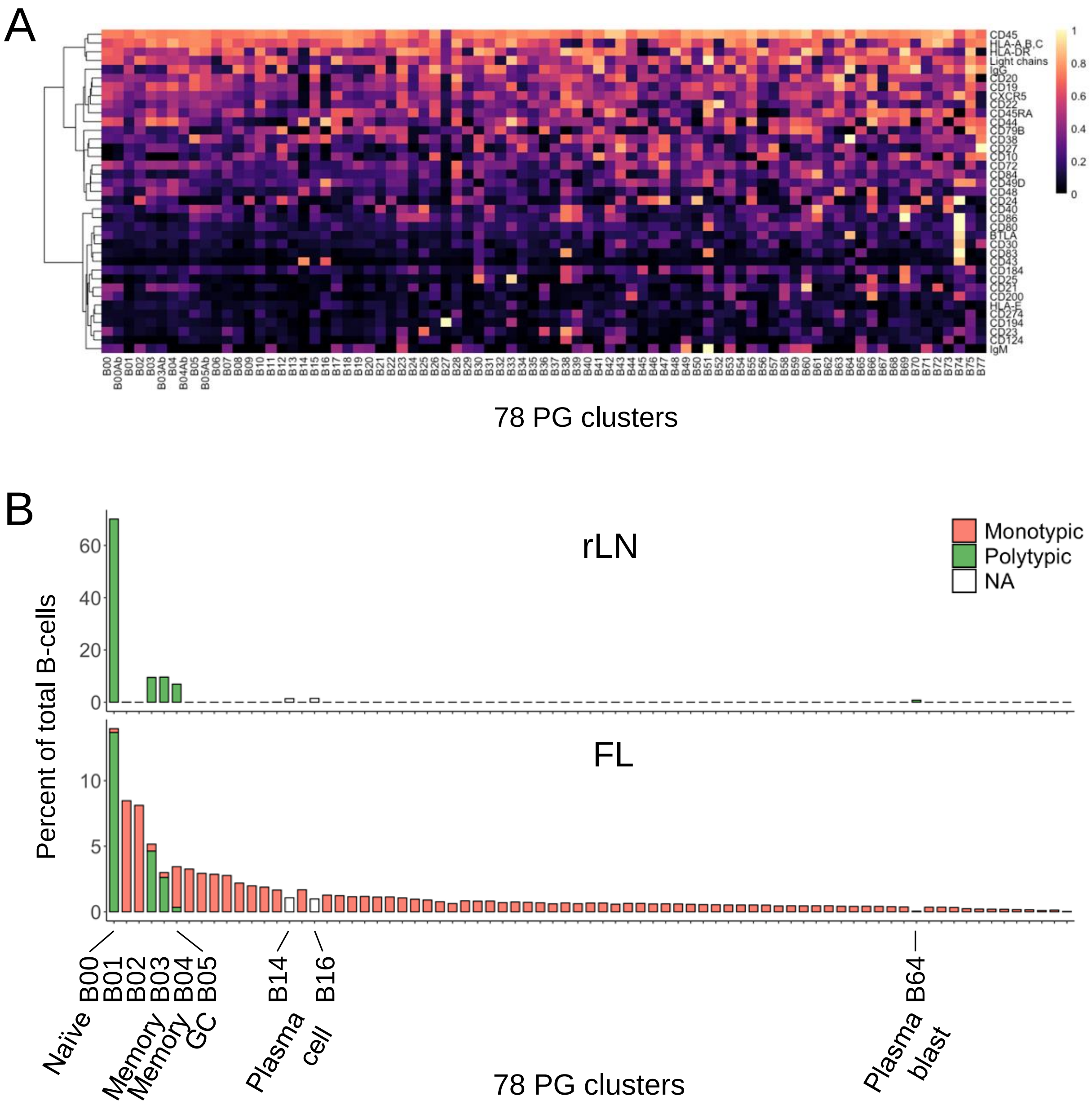

Supplementary Figure 2. Phenograph (PG) clustering of B-cells

A) Protein marker expression heatmap. Expression levels are scaled globally from 0 to 1.
B) Histogram plots of B-cell numbers assigned to each of the 78 PG clusters in rLN and FL samples. Clustering was performed on data from the B-cell panel only. Ordinal numbering of PG clusters was based on cell abundance across the entire dataset. Clusters B00 (naïve B), B03 (IgM+ memory B), B04 (IgG+ memory B), B05 (germinal center B), B14/16 (plasma cells), and B64 (plasmablasts) were identified as the major normal B-cell populations in rLN samples.
PG cluster B27 was excluded from downstream analyses as the cells could not be positively confirmed as of B-lineage by expression of either CD19, CD20, or CD22.

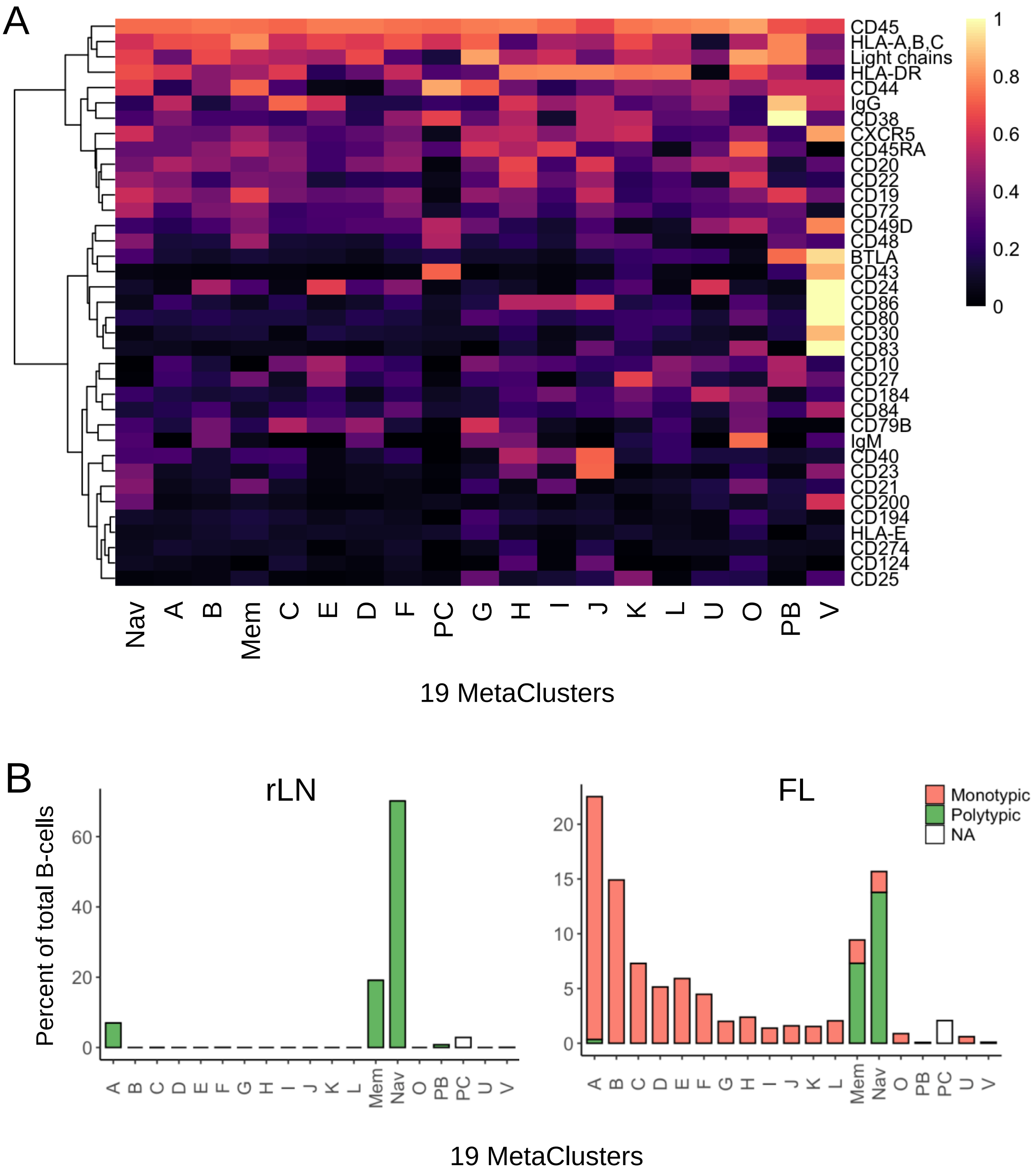

Supplementary Figure 3. Meta-clustering of B-cell subsets

Hierarchical clustering of the 78 B-cell Phenograph clusters yielded 19 MetaClusters.
A) Protein marker expression heatmap. Expression levels are scaled globally from 0 to 1.
B) Histogram plots of B-cell numbers assigned to each of the 19 MetaClusters in rLN and FL samples.

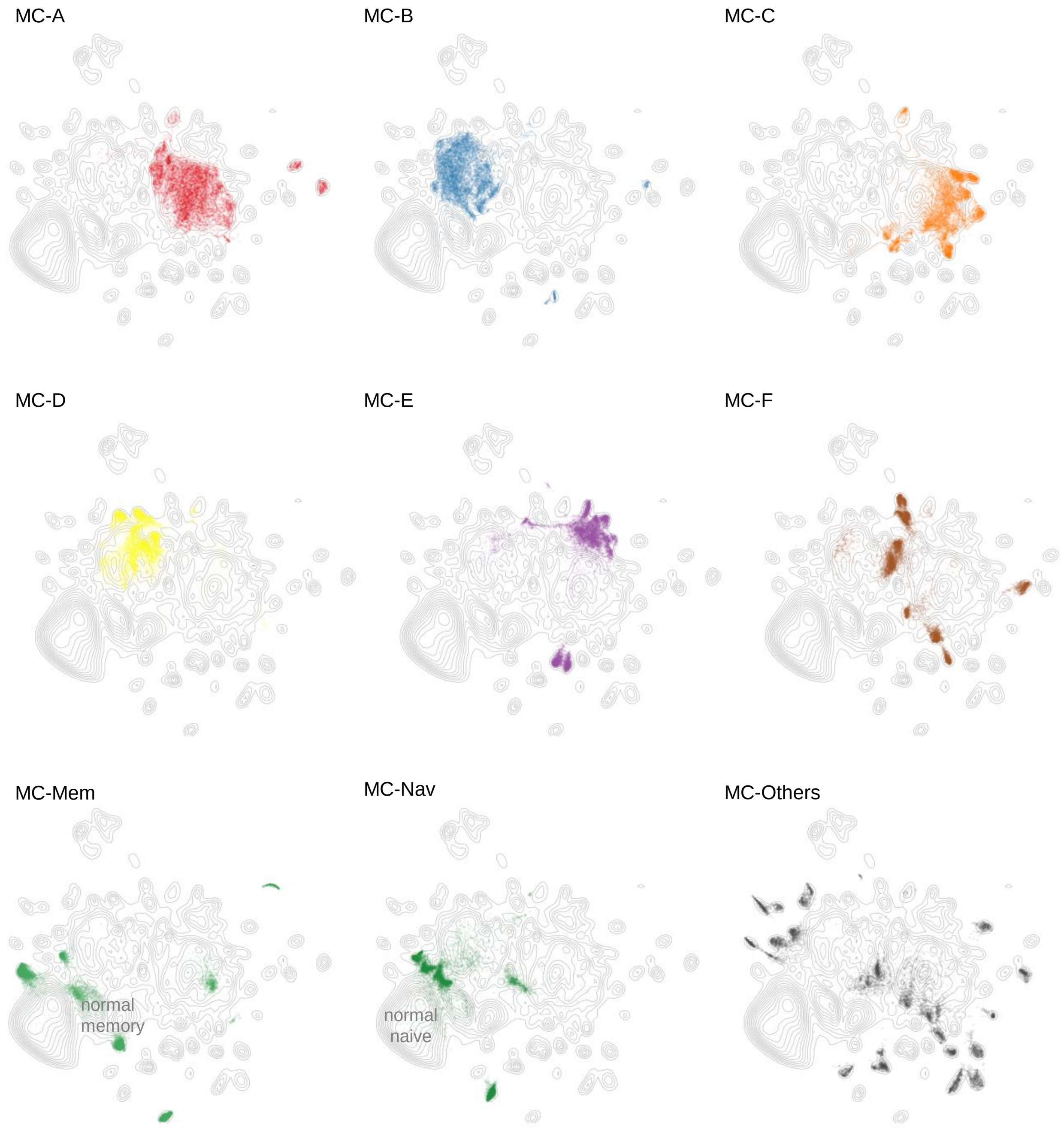

Supplementary Figure 4. UMAP plot of each of the major MetaCluster (MC) groups

The top 6 most abundantly populated MC clusters (A-F) plus Mem and Nav groups are shown separately. Group “Others” includes MC groups G, H, I, J, K, L, O, PB, U, and V. Each dot represents a single cell. Contour lines show density in the combined dataset.

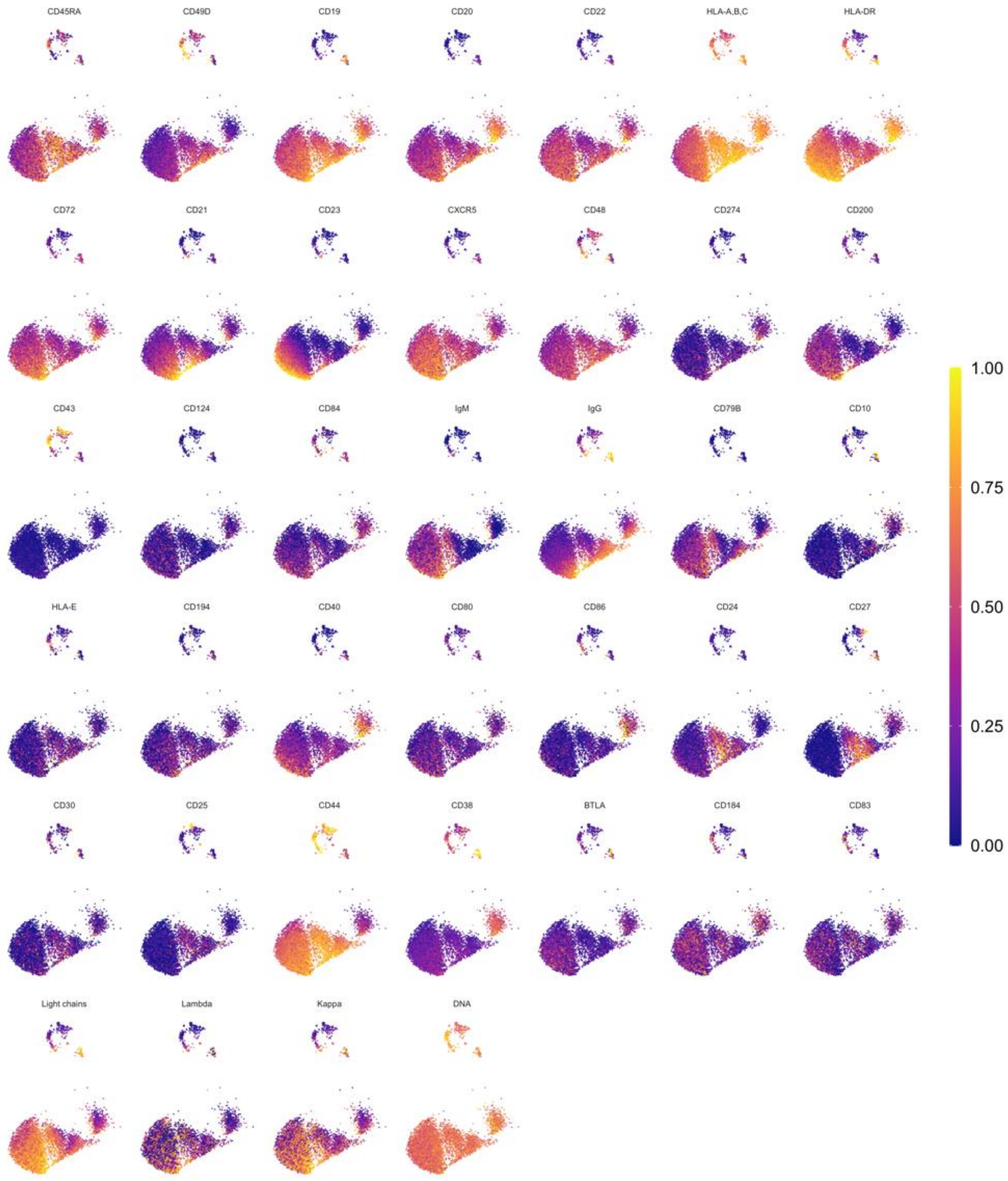

Supplementary Figure 5a. Heatmap of protein marker expression plotted in UMAP space – 36 rLN

Data from the B-cell panel only is depicted. Each dot represents a single cell. Expression levels are scaled for each protein marker.

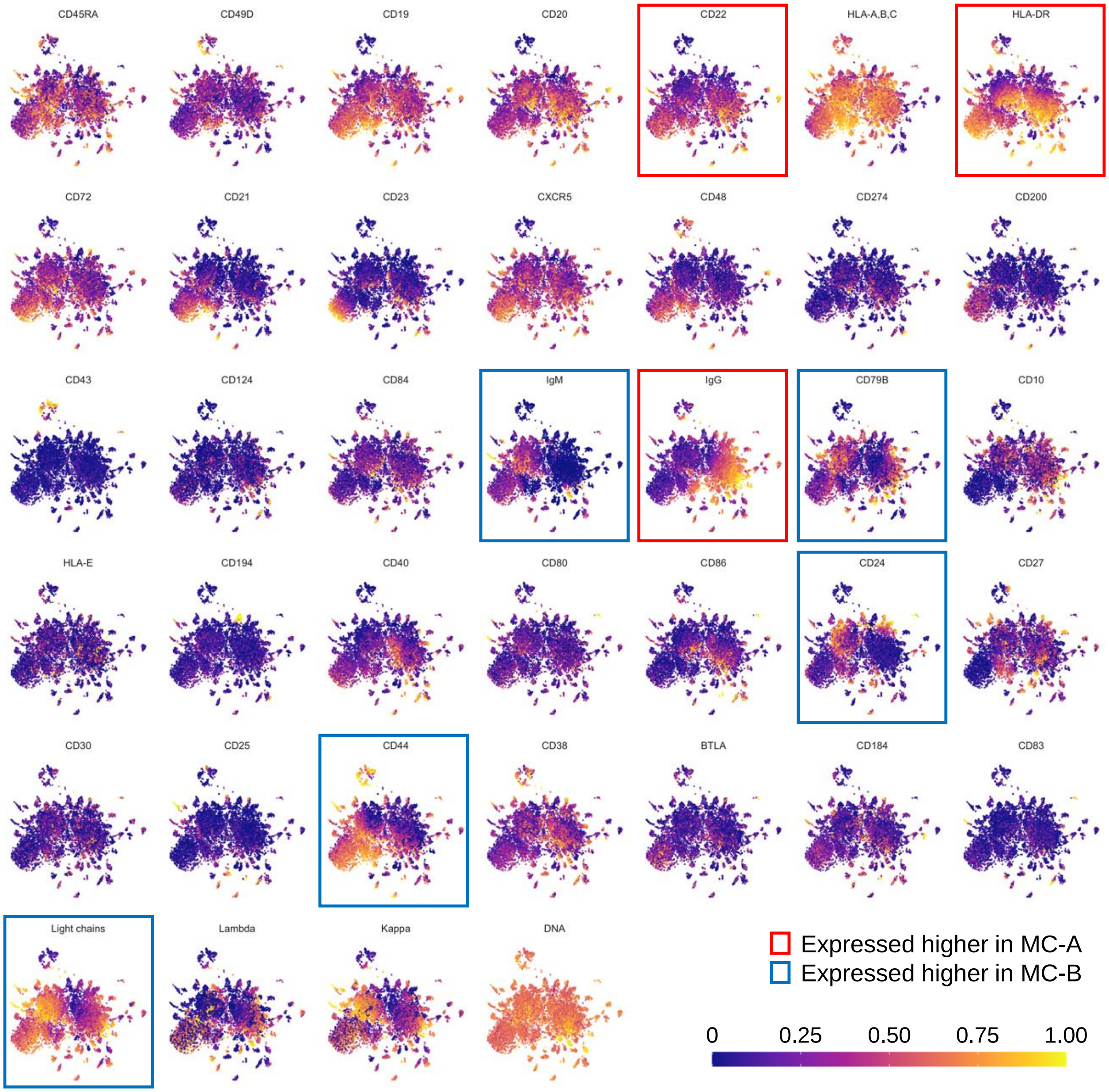

Supplementary Figure 5b. Heatmap of protein marker expression plotted in UMAP space – 154 FL

Data from the B-cell panel only is depicted. Each dot represents a single cell. Expression levels are scaled for each protein marker. Markers contributing at least 2% of total variance to the MC-A vs. MC-B distinction are highlighted.

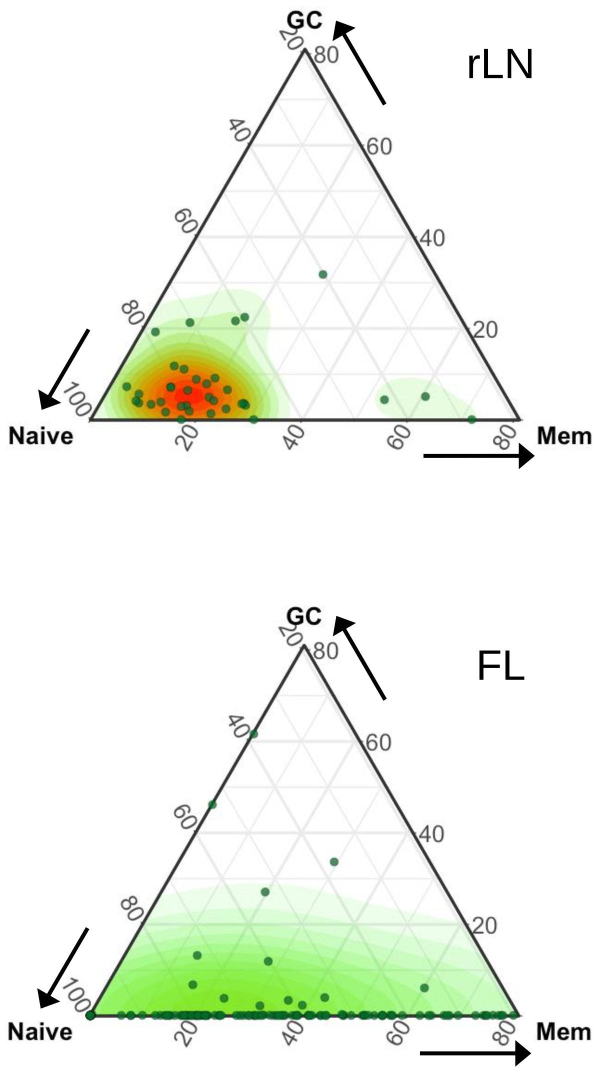

Supplementary Figure 6. Relative proportions of normal B-cells are distorted in FL

Ternary plots depicting relative abundances of normal naïve, memory (Mem), and germinal center (GC) B-cells present within each patient sample. Each dot is a different patient sample.

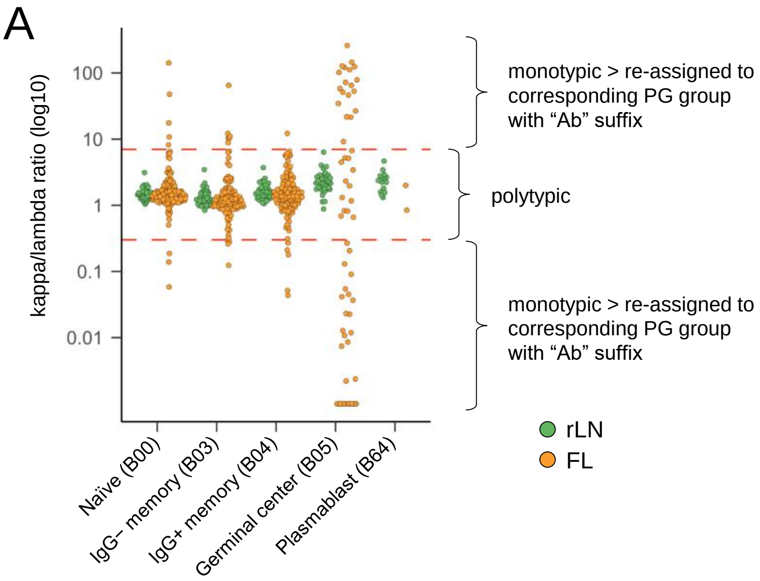

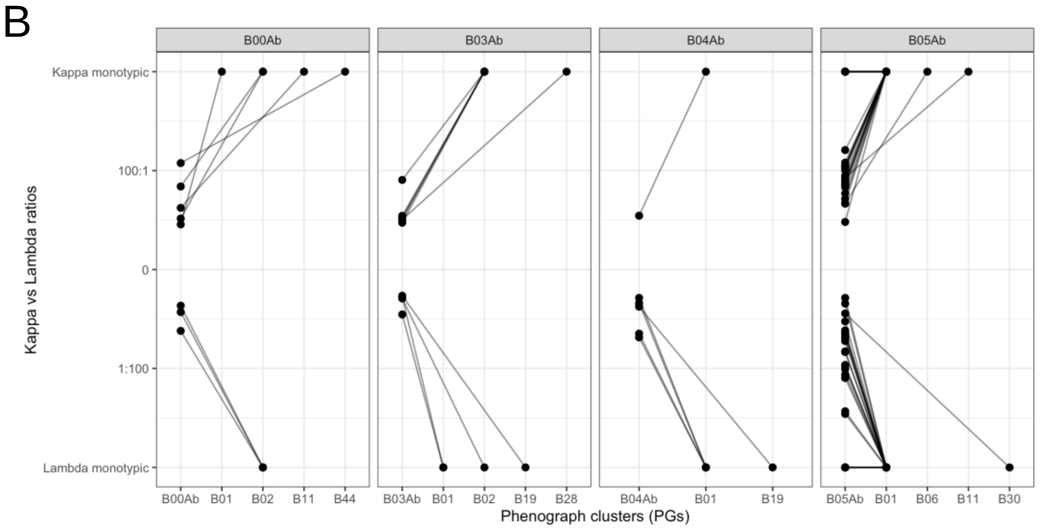

Supplementary Figure 7. B-cell κ/λ ratios in "normal" PG clusters

A) Distribution of observed κ/λ ratios. Each dot represents a different patient sample. Dotted red lines indicate arbitrary thresholds (κ/λ ratio = 7.0 and 0.3). B-cell populations with κ/λ ratios between 7.0 and 0.3 were deemed polytypic, while those outside this range were deemed monotypic and re-assigned to the corresponding PG group with "Ab" suffix (e.g. B05Ab). Naïve (B00), *n*=36 rLN and 140 FL samples; IgG- memory (B03), *n*=34 rLN and 121 FL samples; IgG+ memory (B04), *n*=36 rLN and 104 FL samples; Germinal center (B05), *n*=33 rLN and 62 FL samples; Plasmablast (B64), *n*=15 rLN and 2 FL samples.
B) Observed κ/λ ratios within each of the Ab populations as compared to phenotypically aberrant PG clusters from the same sample. Lines connect B-cell clusters from the same sample. No instances of light chain "mismatch" were observed among the 67 depicted samples of 71 total that contained Ab populations. The remaining 4 could not be assessed in this manner due to indeterminant light chain staining among cells within the non-Ab PG clusters.

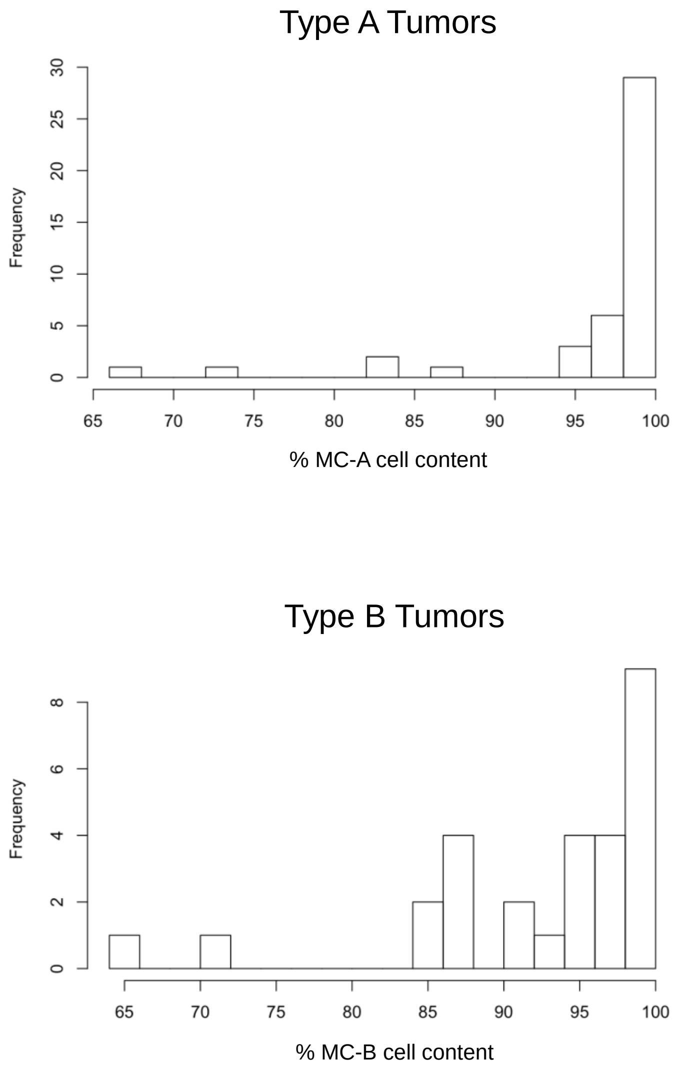

Supplementary Figure 8. MC-A and MC-B cell content of tumors assigned to types A and B, respectively

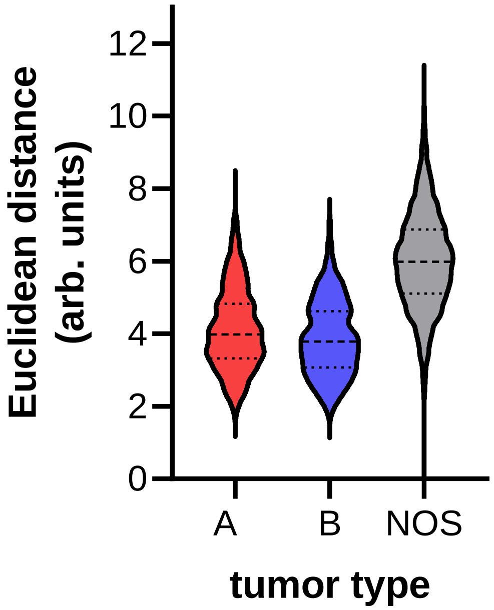

Supplementary Figure 9. Inter-sample pairwise distances

Each FL sample was expressed as a single point in 39-dimensional space based on the median expression value for each of the 39 CyTOF markers. All possible inter-sample pairwise Euclidean distances were then calculated within each sample type category (A vs. B vs. NOS). Dashed lines indicate median values, dotted lines indicate 1st and 3rd quartile values.

NOS, not otherwise specified.

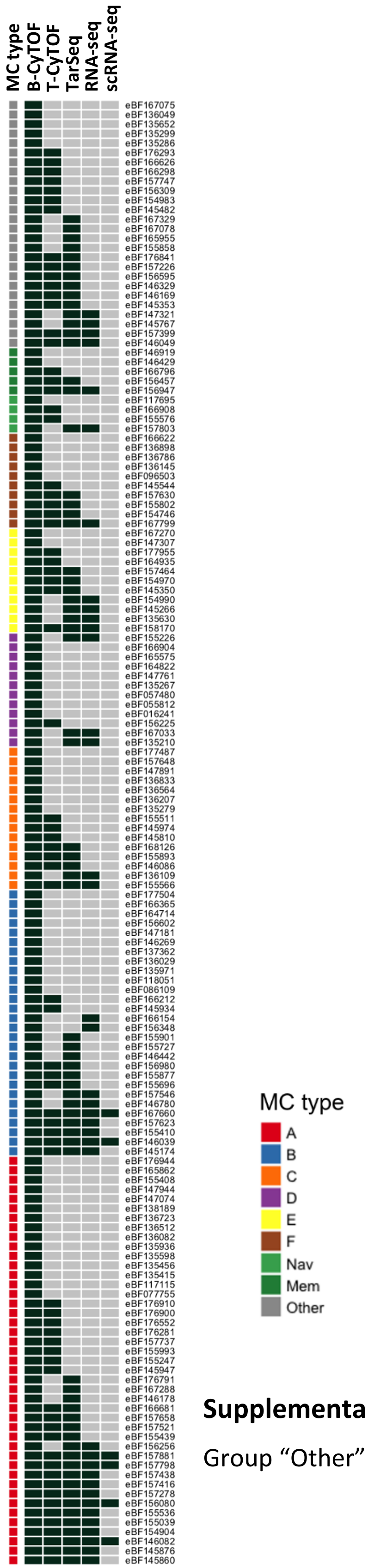

Supplementary Figure 10. Summary of available data types for each sample

Group "Other" includes Types G, H, I, J, K, L, O, PB, U, and V.

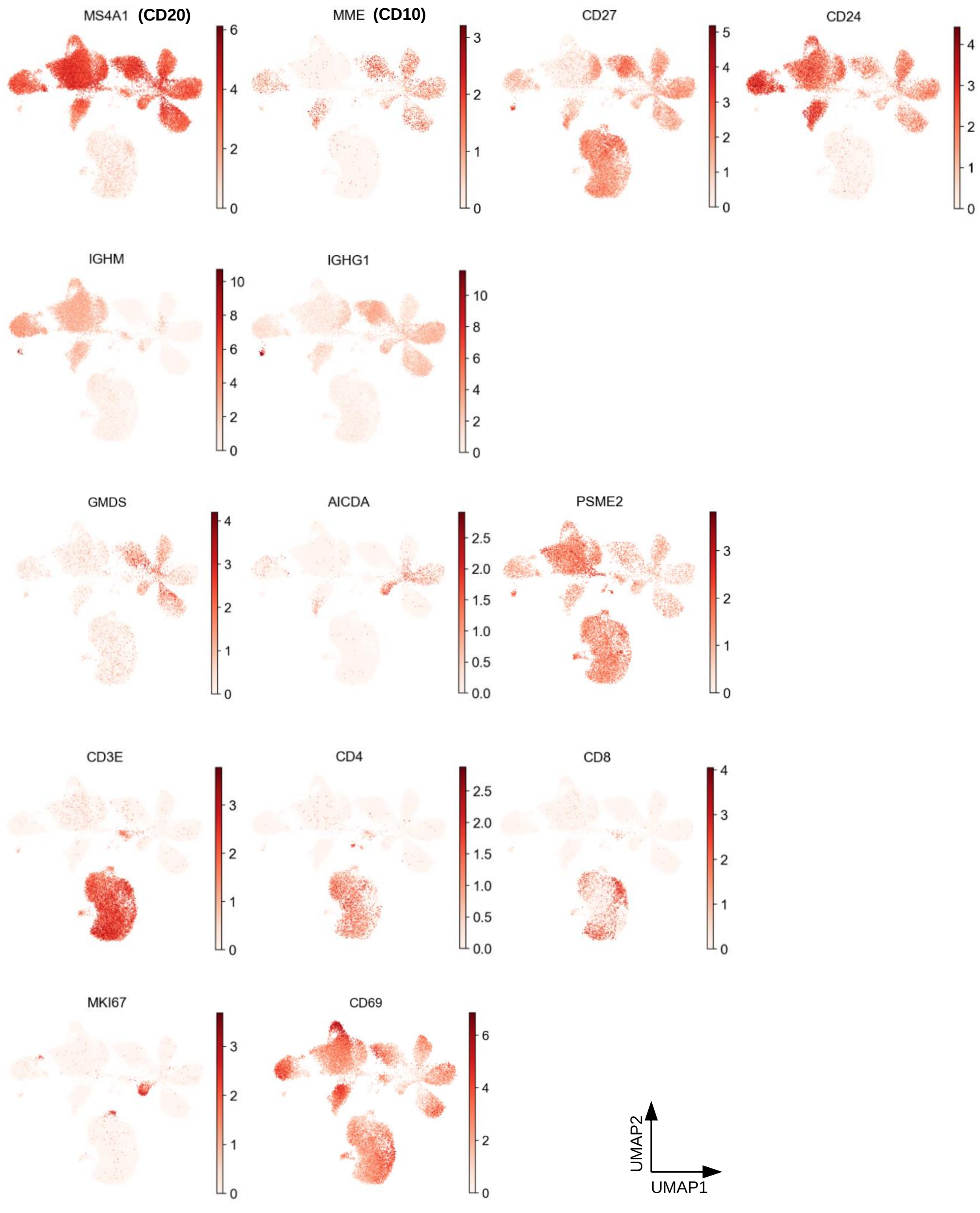

Supplementary Figure 11. RNA expression levels from scRNA-seq data

Plots contain all 6 FL + 4 rLN samples. Expression heatmaps show log-normalized counts. Selected individual marker genes are depicted.

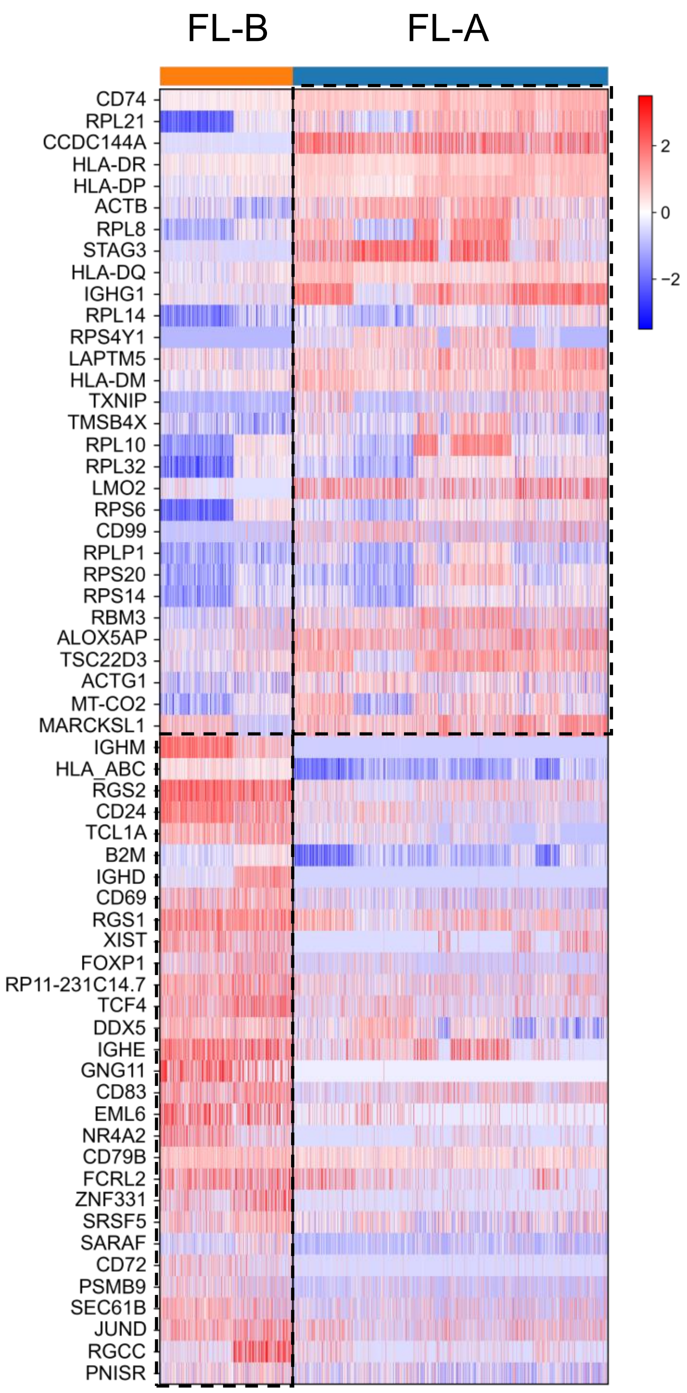

Supplementary Figure 12. Top genes differentially expressed between abnormal B-cells from type A vs. B FL samples using scRNA-seq data

Gene expression heatmap of 7916 and 3337 abnormal B-cells from 4 type A and 2 type B FL samples, respectively, selected from the CyTOF cohort. The top 30 differentially expressed genes from each set of cells as ranked by adjusted p-value after filtering for log2 fold-change > 1 are depicted (*padj<.05; 2-sided Wilcoxon test with BH correction*). See **Supplementary Data 6**. Expression values are log-normalized counts (mean 0, sd 1).

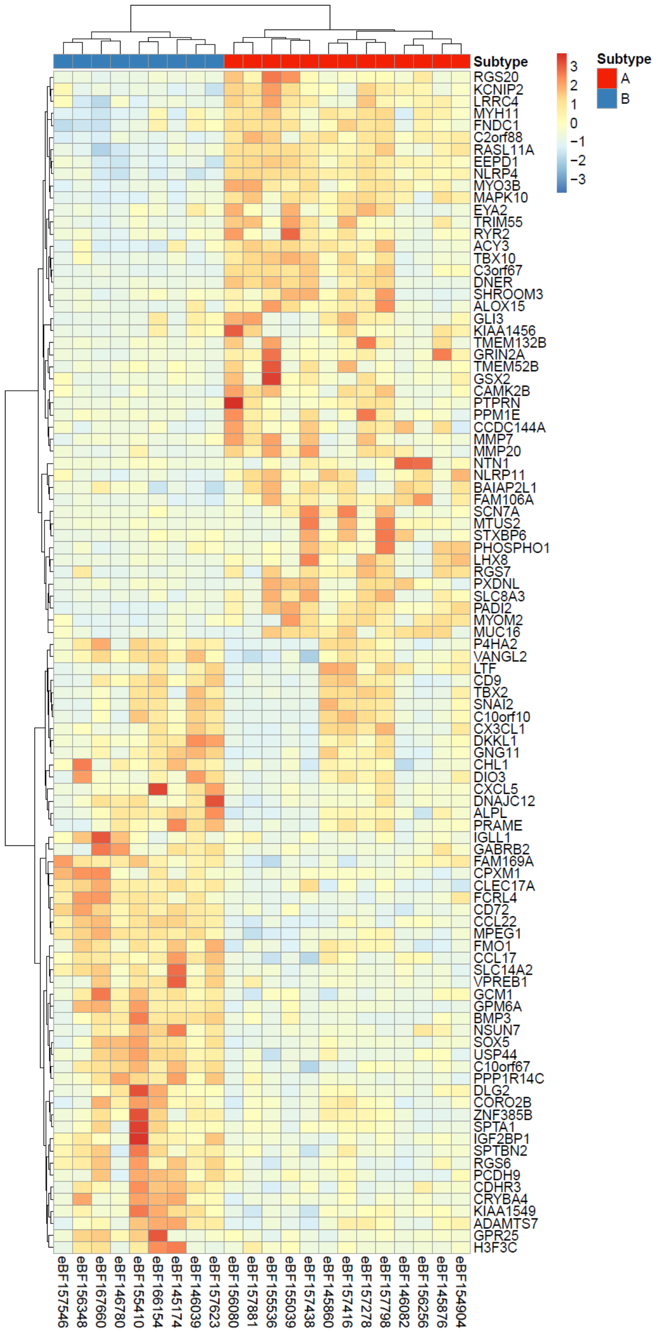

Supplementary Figure 13. Top genes differentially expressed between type A vs. B FL samples using bulk RNA-seq data

Gene expression heatmap of whole tissue or unfractionated cell suspension material from 13 type A and 9 type B FL samples selected from the CyTOF cohort. The top 48 and 50 differentially expressed genes from type A and type B samples, respectively, as ranked by adjusted p-value after filtering for log2 fold-change > 3 are depicted (*padj<.05; 2-sided Wald test with BH correction*). Gene lists using less stringent fold-change cutoffs failed to yield appreciable numbers of significant pathways in Reactome analyses (see **Supplementary Data 6**). Expression values are log-normalized counts (mean 0, sd 1).

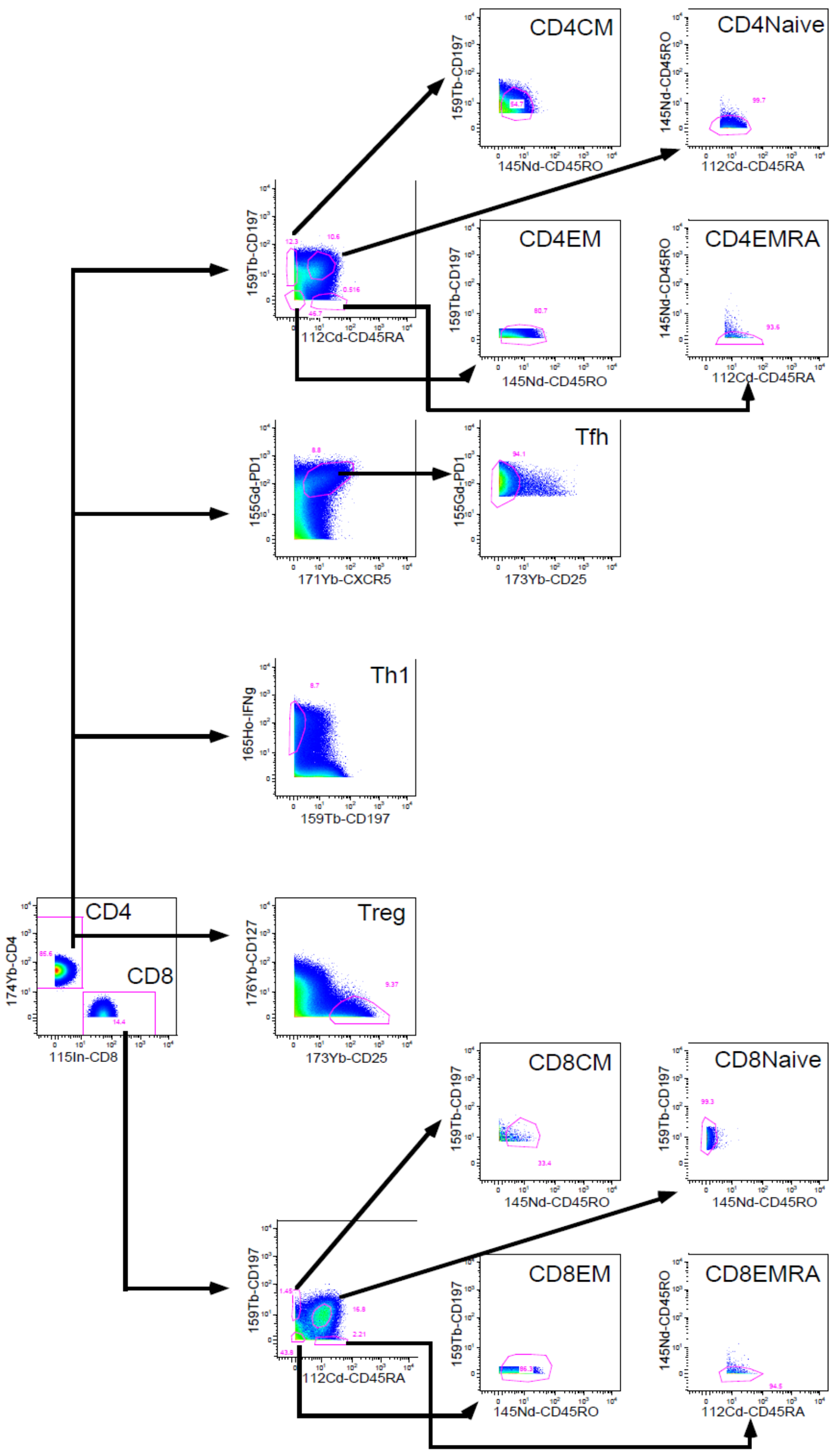

Supplementary Figure 14. Gating strategy for T-cell landmark populations

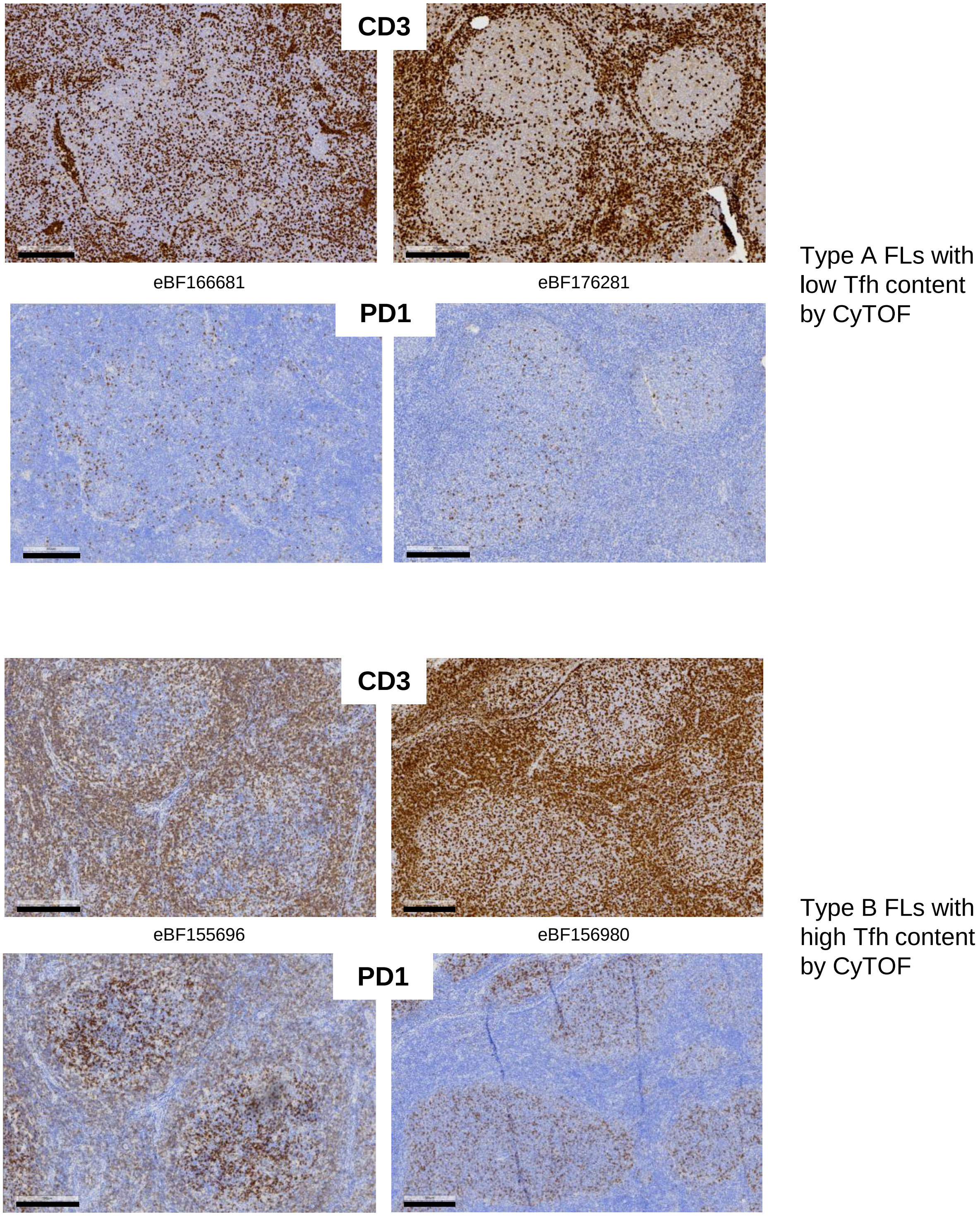

Supplementary Figure 15. Immunohistochemical staining of tissue sections from diagnostic FL samples

Cases were selected for high vs. low Tfh cell content based on CyTOF data. Representative, corresponding fields from serial sections are shown for each patient sample. Whole tissue sections were stained once with the indicated markers. Bar = 300um.

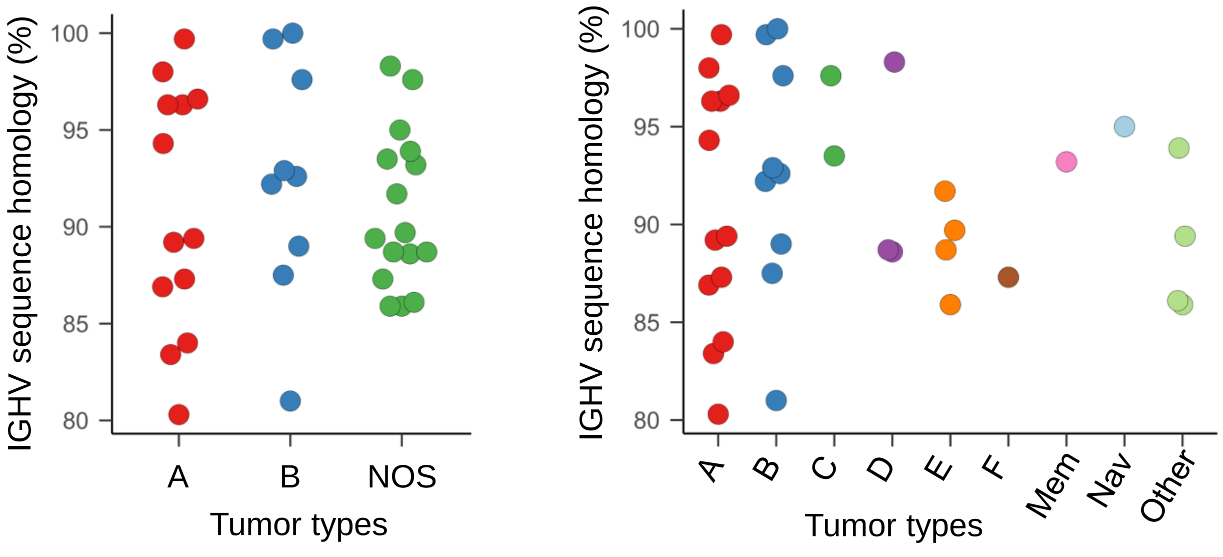

Supplementary Figure 16. Somatic hypermutation (SHM) analysis

IGHV sequence homology based on bulk RNA-seq data (*n*=38). Each dot represents an individual FL sample, colored by tumor MC type.

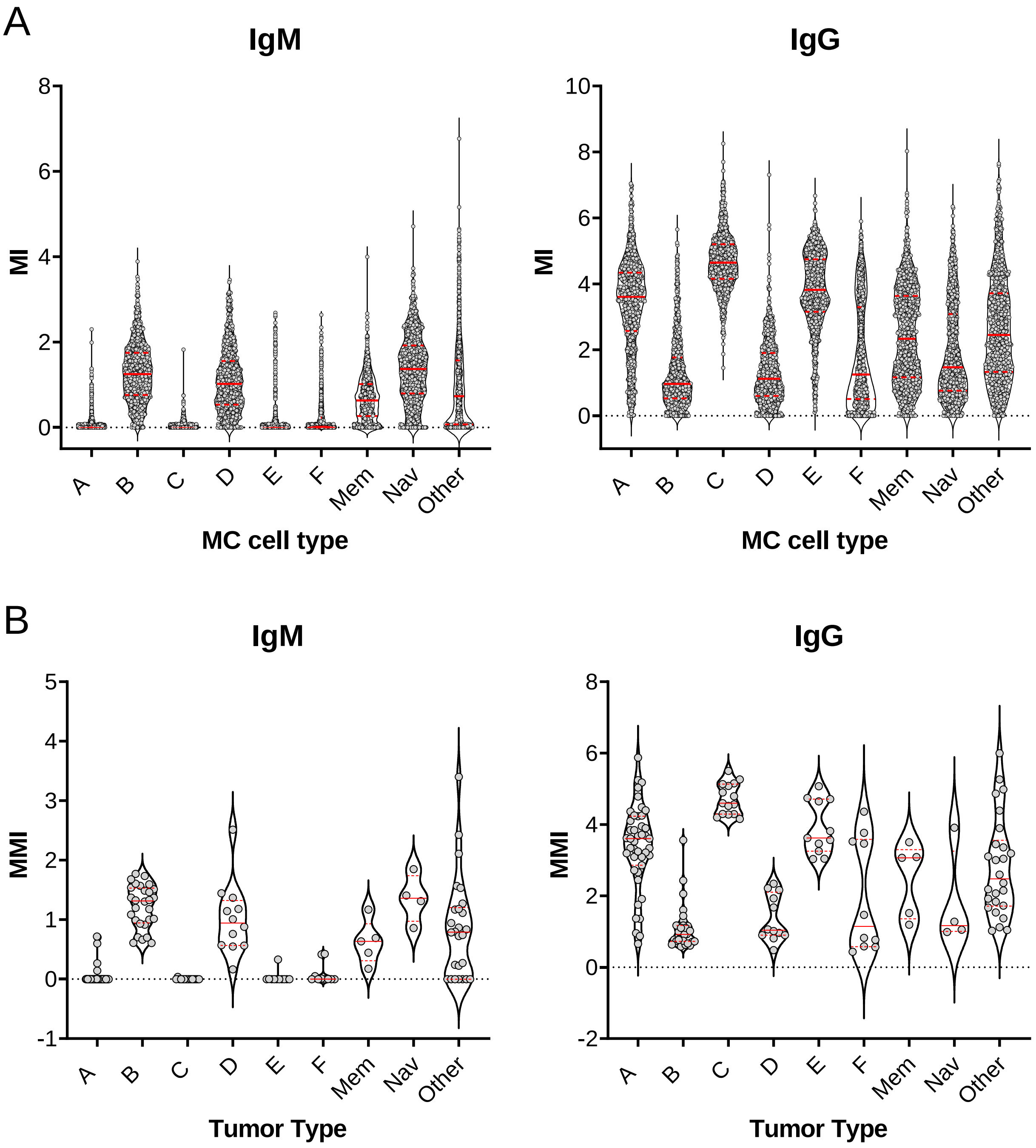

Supplementary Figure 17. IgM/IgG protein expression level by CyTOF

A) Expression per cell, grouped by MC cluster

B) Mean expression per sample, grouped by tumor MC type

MI, mass intensity; MMI, mean mass intensity

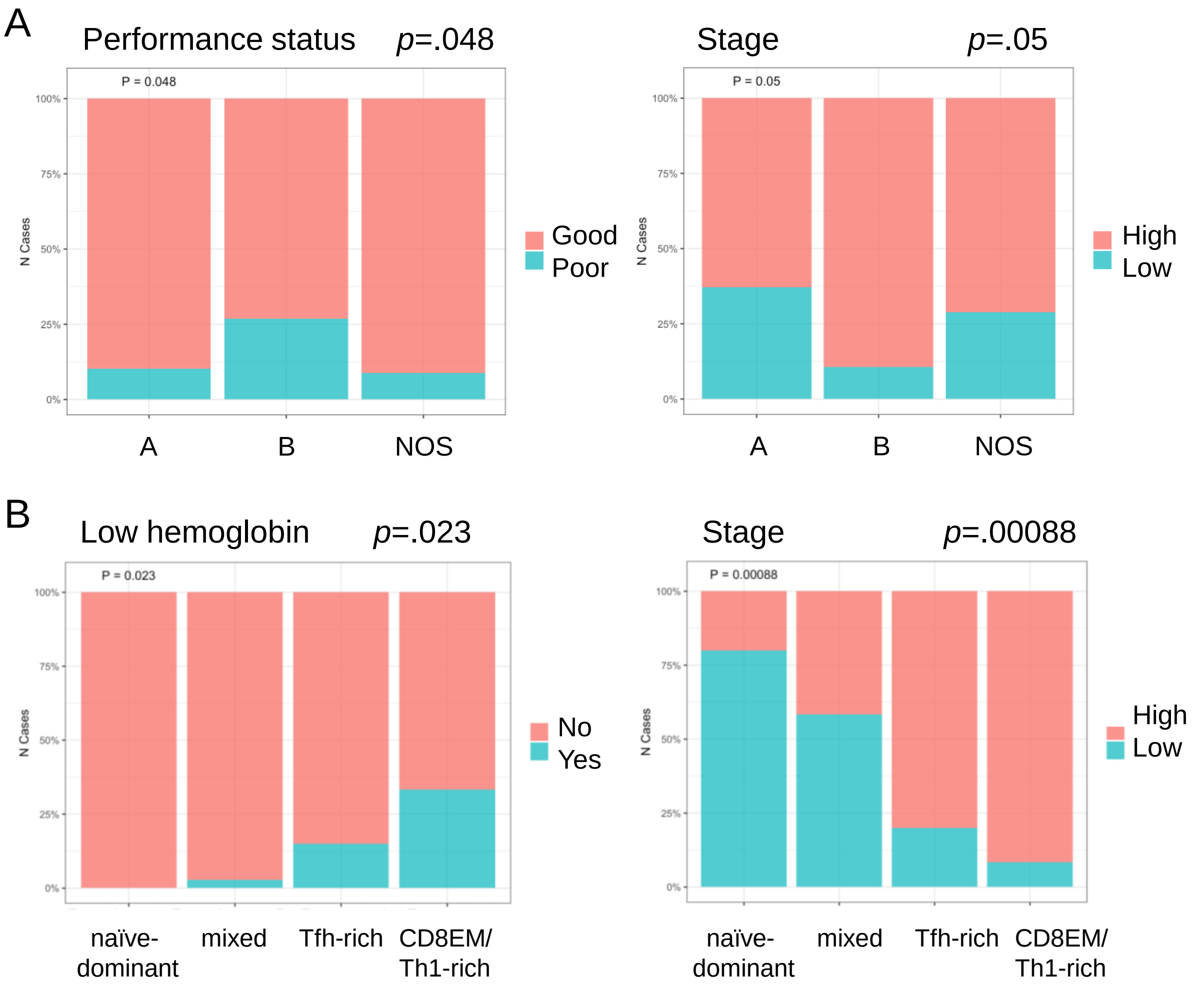

Supplementary Figure 18. Baseline clinical feature correlations

Features correlated with **A**) tumor MC type and **B**) T-cell signature. Only significant correlations are shown (Chi-square test, 1-sided). The indicated *p*-values were not adjusted for multiple testing.

NOS, not otherwise specified.

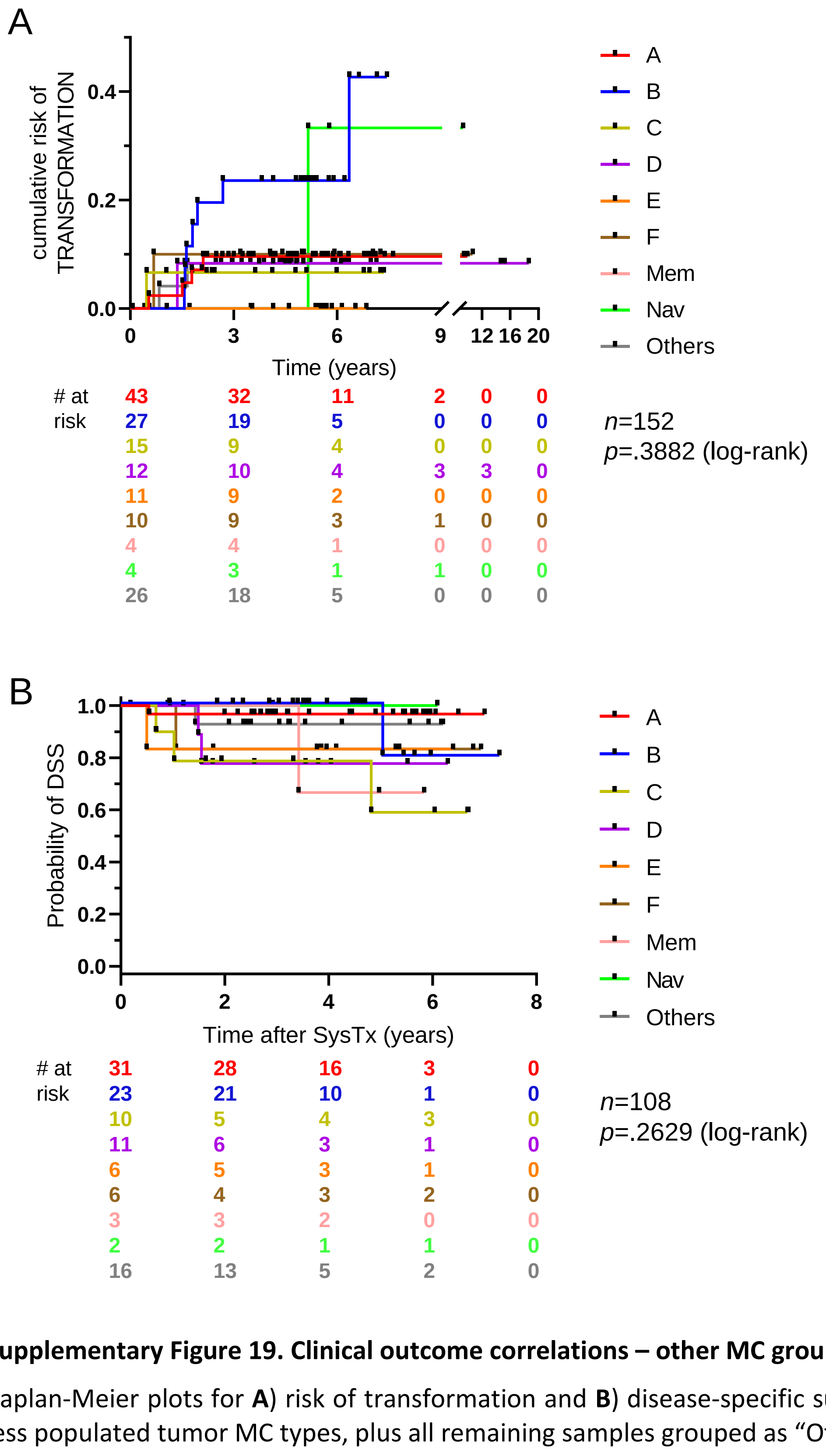

Supplementary Figure 19. Clinical outcome correlations – other MC groups

Kaplan-Meier plots for **A**) risk of transformation and **B**) disease-specific survival (DSS) including less populated tumor MC types, plus all remaining samples grouped as “Others”.
SysTx, systemic therapy.

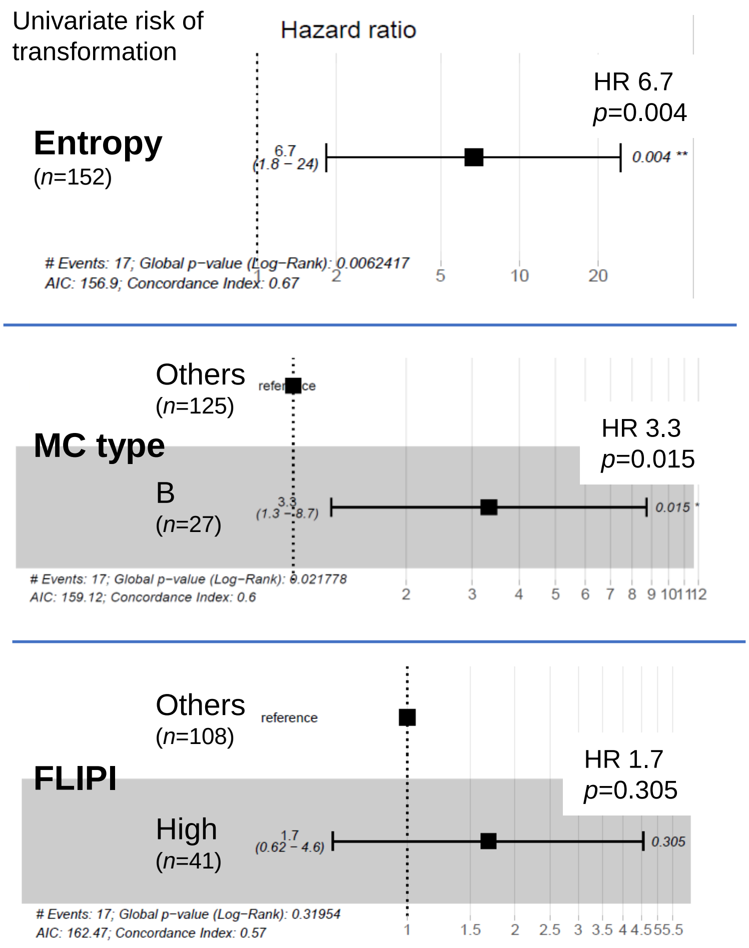

Supplementary Figure 20. Clinical outcome correlations – risk of transformation

Forest plot of univariate features for risk of transformation by Cox proportional hazards model. Hazard ratios (HR) are plotted with error bars indicating 95% CI.

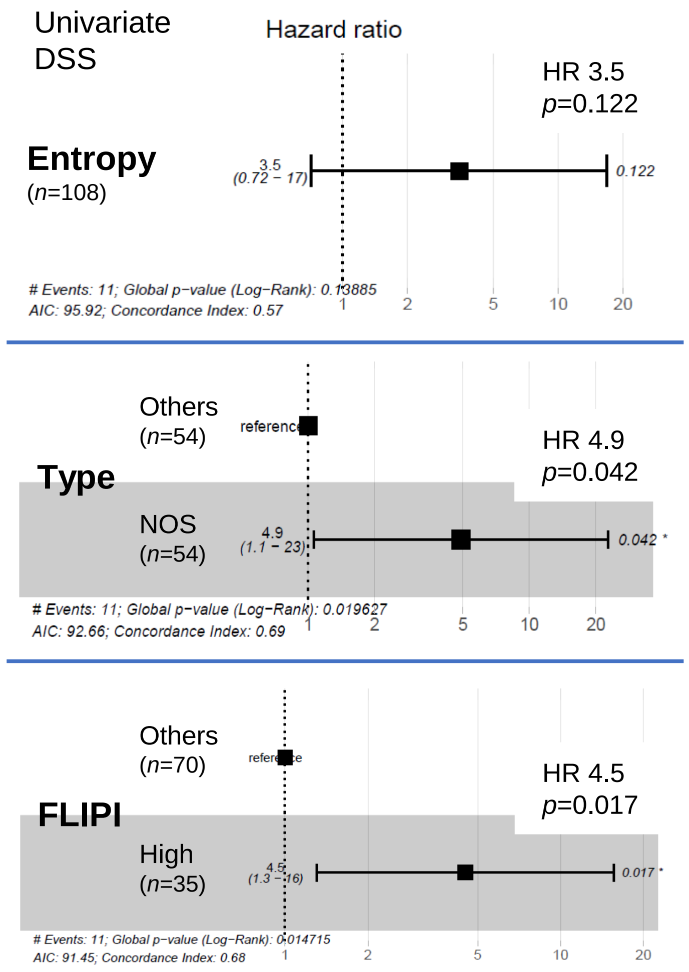

Supplementary Figure 21. Clinical outcome correlations – disease specific survival (DSS)

Forest plot of univariate features for DSS by Cox regression model. Entropy is taken as a continuous variable, while tumor MC type (NOS vs other) and FLIPI score (High vs. others) are each divided into 2 categories. Hazard ratios (HR) are plotted with error bars indicating 95% CI.

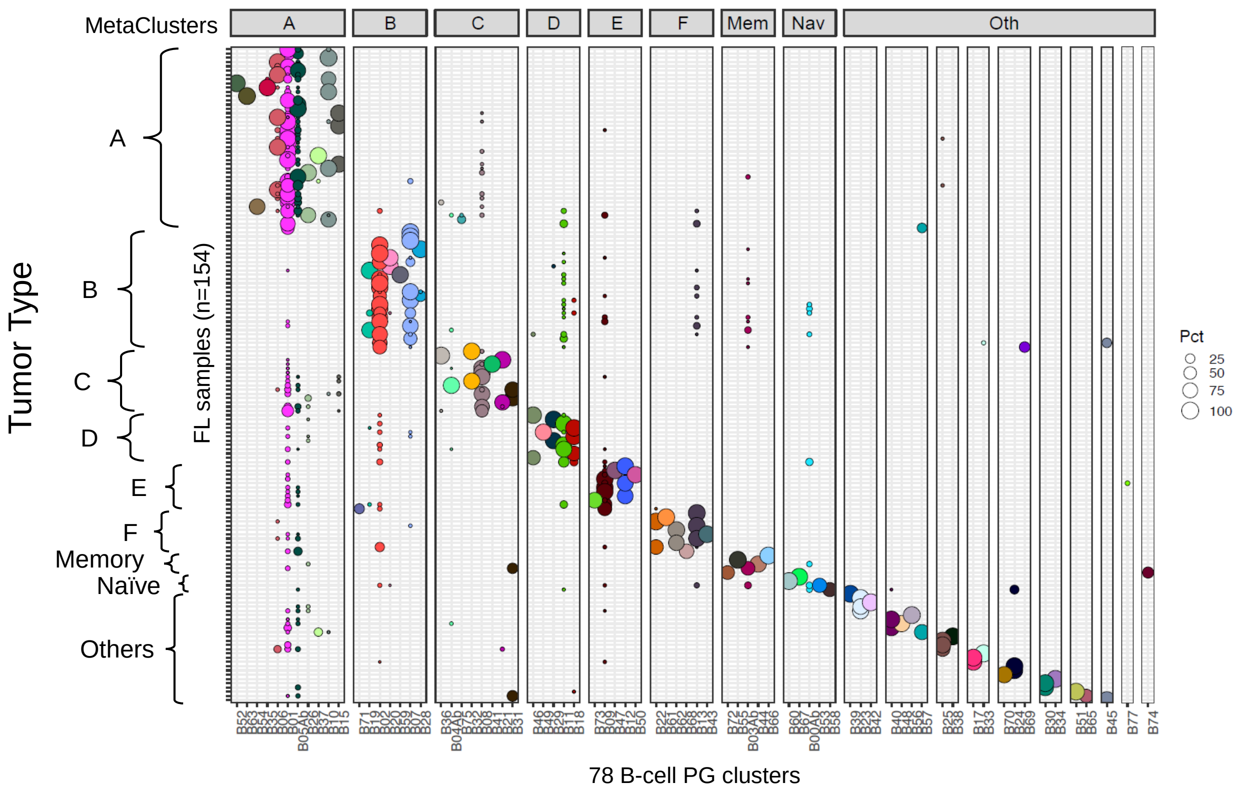

Supplementary Figure 22. Composition of FL samples according to B-cell PG clusters

Colored bubbles within each row (= sample) indicate cells assigned to a given PG cluster (=column) with size proportional to their relative abundance in the sample (each row adding up to 100%). PG clusters are grouped into metacluster (MC) groups. Samples are grouped by tumor type, which is defined as being composed of greater than 50% tumoral B-cells of the corresponding MC type.

This figure is identical to **Fig 3d**, but with re-ordering of FL samples by tumor type rather than by MC-A/B cell content.

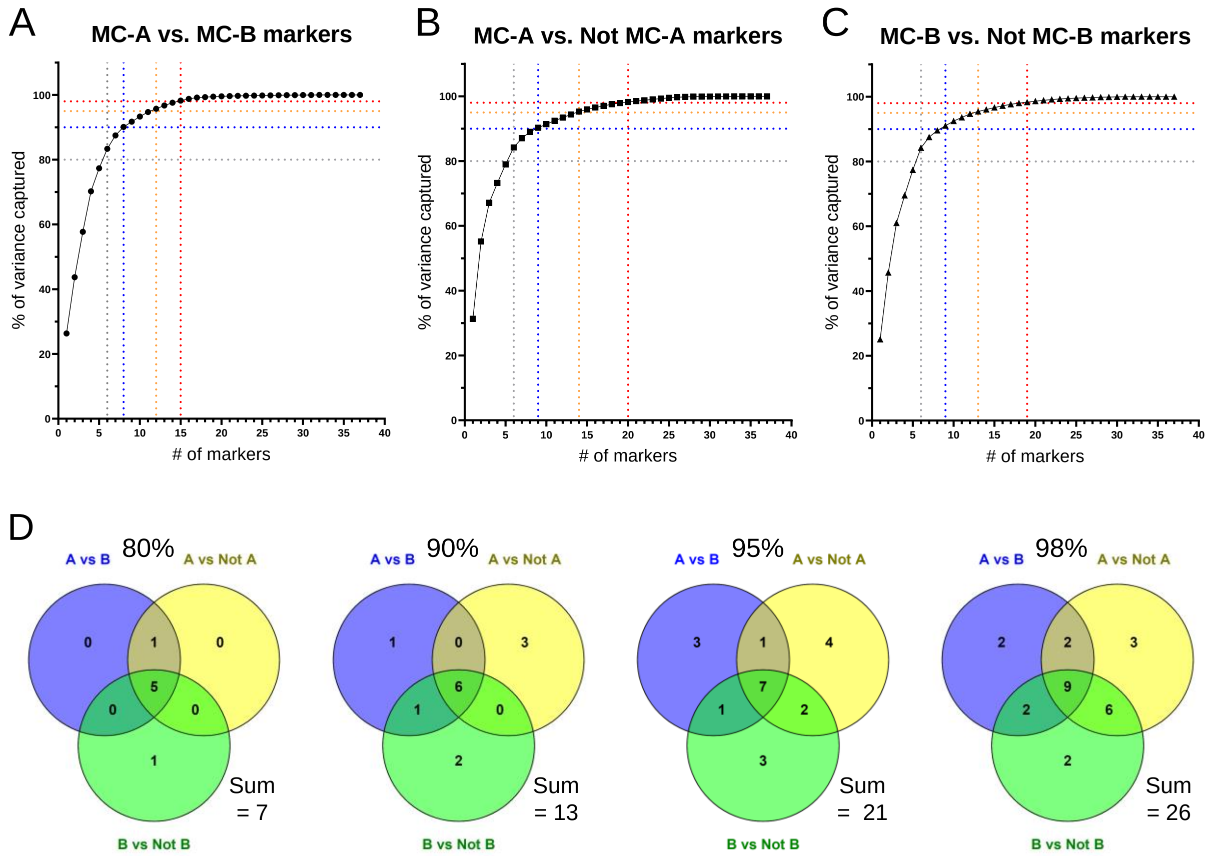

Supplementary Figure 23. Information content plots

Cumulative variance in marker contribution to comparisons
A) MC-A vs. MC-B, **B**) MC-A vs. Not MC-A, and **C**) MC-B vs. Not MC-B, is plotted on the Y-axis. Markers along the X-axis are listed in **Supplementary Data 4**. Dotted lines highlight 80%, 90%, 95%, and 98% cumulative variance thresholds.
D) Venn diagrams indicating overlap among markers in panels A-C at each of 80%, 90%, 95%, and 98% thresholds. The 26-marker panel which retains 98% of information content is listed in **Supplementary Data 9**.

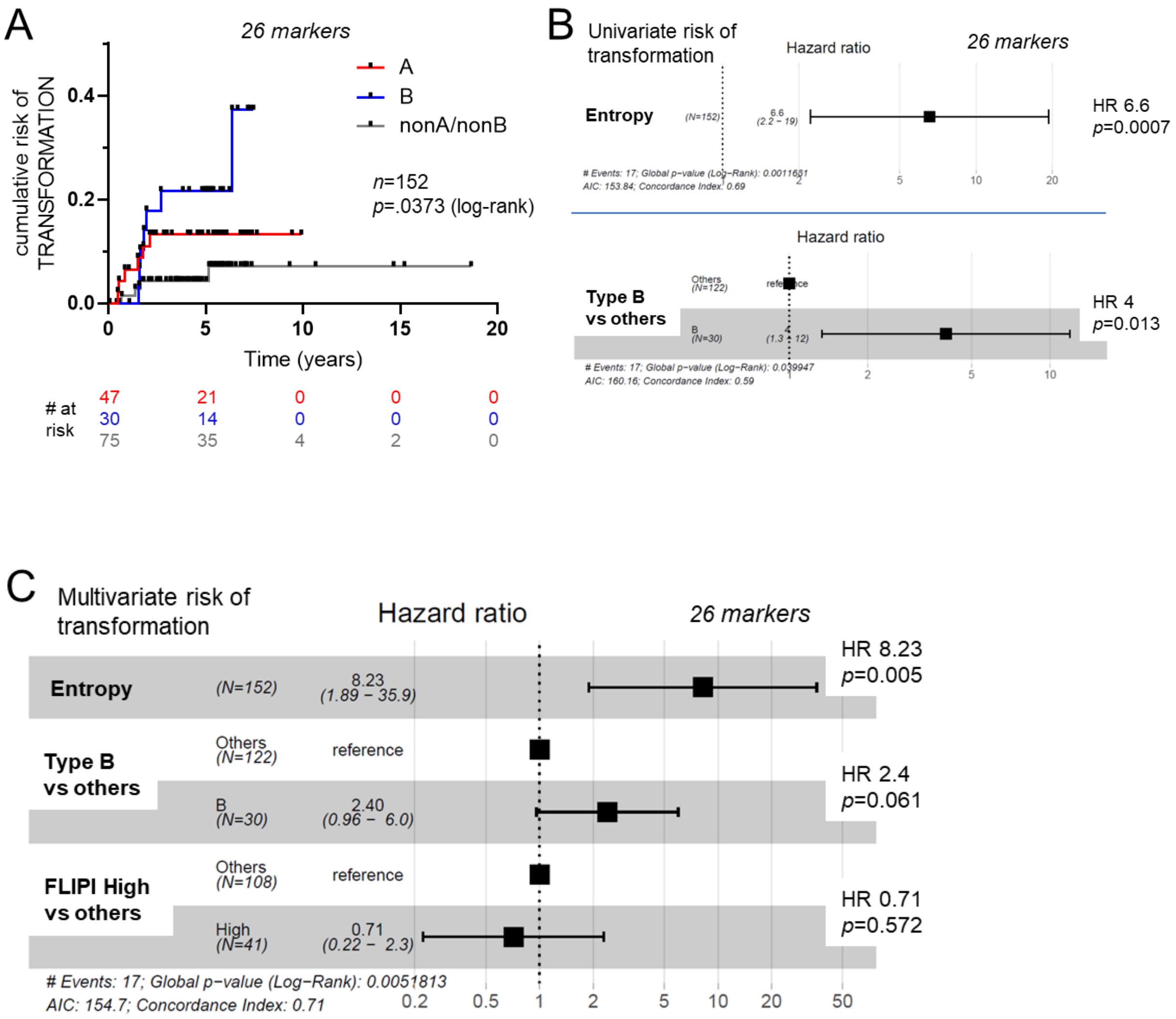

Supplementary Figure 24. Clinical outcome correlation – risk of transformation using a reduced 26-marker CyTOF panel

A) Kaplan-Meier curve for transformation risk by 3 MC groups.
B,C) Forest plots from **B**) univariate and **C**) multivariate analyses of transformation risk by Cox (univariate Entropy) and weighted Cox (univariate MC type and multivariate) regression models. Entropy is taken as a continuous variable, while tumor MC type (Type B vs. others) and FLIPI score (High vs. others) are each divided into 2 categories. Hazard ratios (HR) are plotted with error bars indicating 95% CI.

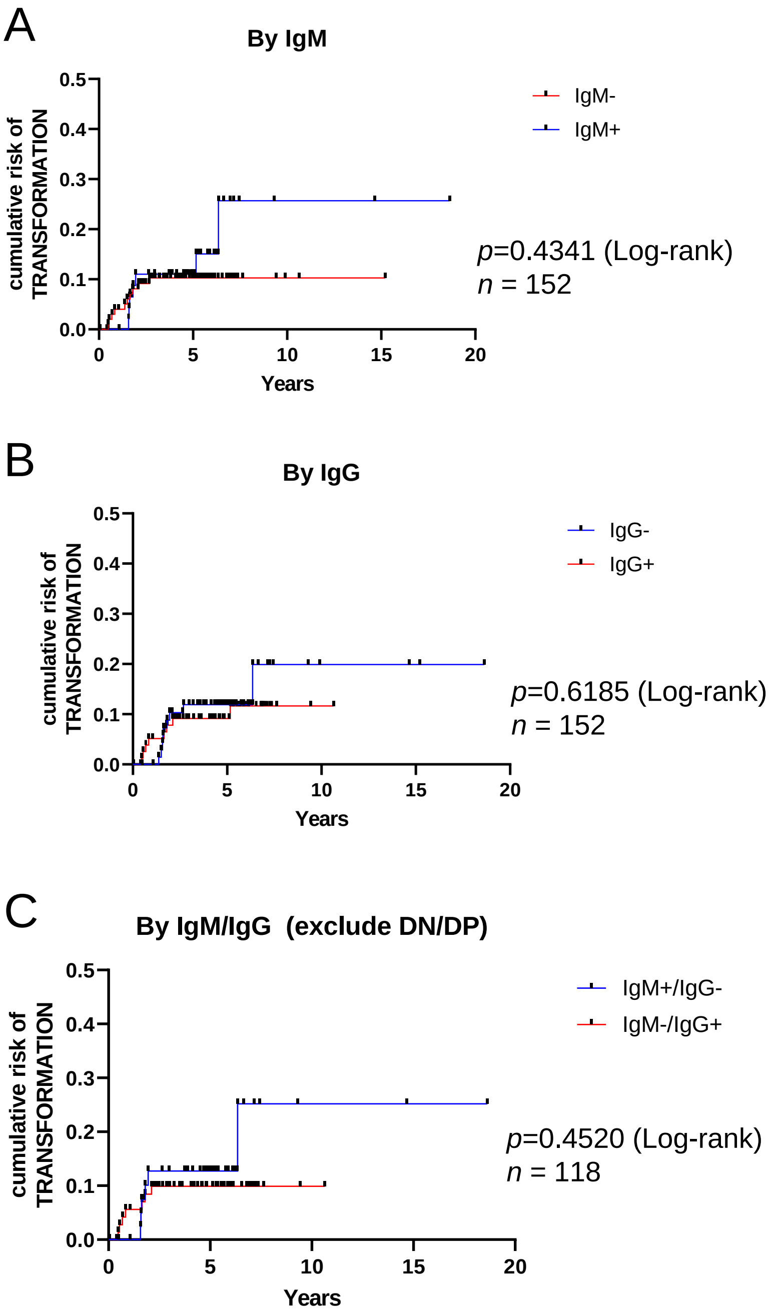

Supplementary Figure 25. Kaplan-Meier plots for risk of transformation by IgM/IgG status

Binary segregation of patient FL samples by **A**) IgM positive vs. negative, **B**) IgG positive vs. negative, and **C**) IgM positive/IgG negative vs. IgM negative/IgG positive. In **C**, samples scored as IgM+/IgG+ (double positive, or DP) or IgM-/IgG- (double negative, or DN) were excluded from analysis. The actual distribution of expression values for each marker was used to define positive/negative thresholds (*k*-means clustering).

**CyTOF FL cohort (*n*=154)**	**No.**	**%**	*p*-value	**All FL over same time period (*n*=992)**	**No.**	**%**
Age (range 15-89; median 59)			0.0001	Age (range 24-104; median 64)		
≤60	88	57.1		≤60	380	38.3
>60	66	42.9		>60	612	61.7
Gender			1	Gender		
Female	72	46.8		Female	464	46.8
Male	82	53.2		Male	528	53.2
Performance Status			0.1689	Performance Status		
0	42	29.2		0	285	32.2
1	84	58.3		1	452	62.5
2	16	11.1		2	103	11.7
3	2	1.4		3	37	4.2
4	0	0.0		4	6	0.6
N/A				N/A	109	
B symptoms			0.5278	B symptoms		
Absent	130	84.4		Absent	800	86.5
Present	24	15.6		Present	125	13.5
N/A				N/A	67	
Stage			0.1948	Stage		
I-II	43	27.9		I-II	317	33.6
III-IV	111	72.1		III-IV	627	66.4
N/A				N/A	48	
Grade			0.0923	Grade		
1-2	127	82.5		1-2	859	87.7
3	27	17.5		3	120	12.3
N/A				N/A	13	
Tumor mass			0.0263	Tumor mass		
<7 cm	95	65.1		<7 cm	612	74.2
≥7 cm	51	34.9		≥7 cm	213	25.8
N/A	8			N/A	167	
Bone marrow			0.0839	Bone marrow		
Not involved	90	58.4		Not involved	592	65.7
Involved	64	41.6		Involved	309	34.3
N/A				N/A	91	
Extranodal			0.8903	Extranodal		
<2 sites	136	88.3		<2 sites	801	88.8
≥2 sites	18	11.7		≥2 sites	101	11.2
N/A				N/A	90	
Hemoglobin			0.7988	Hemoglobin		
≥120 g/L	134	87.6		≥120 g/L	837	86.3
<120 g/L	19	12.4		<120 g/L	132	13.6
N/A	1			N/A	23	
LDH			0.3322	LDH		
≤ULN	126	81.8		≤ULN	715	85.0
>ULN	28	18.2		>ULN	126	15.0
N/A				N/A	151	
FLIPI			0.3926	FLIPI		
Low	63	41.7		Low	234	38.7
Intermediate	46	30.5		Intermediate	220	36.4
High	42	27.8		High	151	25.0
N/A	3			N/A	387	
Primary Treatment			0.0134	Primary Treatment		
Observation or local treatment	39	25.5		Observation or local treatment	353	35.8
Systemic treatment	114	74.5		Systemic treatment	631	64.1
NA	1			NA	8	

Supplementary Table 1. Clinical characteristics at baseline

Statistical *p*-values were calculated by Fisher's exact test (2-sided) for two category variables or Chi-square test (2-sided) for >2 variables. Performance status *p*-value was calculated for 4 categories (0, 1, 2, and 3/4). The indicated *p*-values were not adjusted for multiple testing.

	**Tumor MC type (*p*-value)**	**T-cell signature (*p*-value)**
Age	0.4388	0.4463
≤60		
>60		
Gender	0.0874	0.6660
Female		
Male		
Performance Status	0.0478	0.5532
0-1		
2-3		
B symptoms	0.3140	0.3304
Absent		
Present		
Stage	0.0497	0.0009
I-II		
III-IV		
Grade	0.3211	0.9441
1-2		
3		
Tumor mass	0.7789	0.0701
<7 cm		
≥7 cm		
Bone marrow	0.1573	0.3705
Not involved		
Involved		
Extranodal	0.6847	0.3458
<2 sites		
≥2 sites		
Hemoglobin	0.5629	0.0225
≥120 g/L		
<120 g/L		
LDH	0.0818	0.6240
≤ULN		
>ULN		

Supplementary Table 2. Significance tests on baseline characteristics

Chi-square *p*-values (1-sided) are shown without adjustment for multiple testing.
Tumor MC type = Type A vs. B vs. nonA/nonB.
T-cell signature = Naïve-dominant vs. Mixed vs. Tfh-rich vs. CD8EM/Th1-rich.

Uncropped images relating to Figure S15

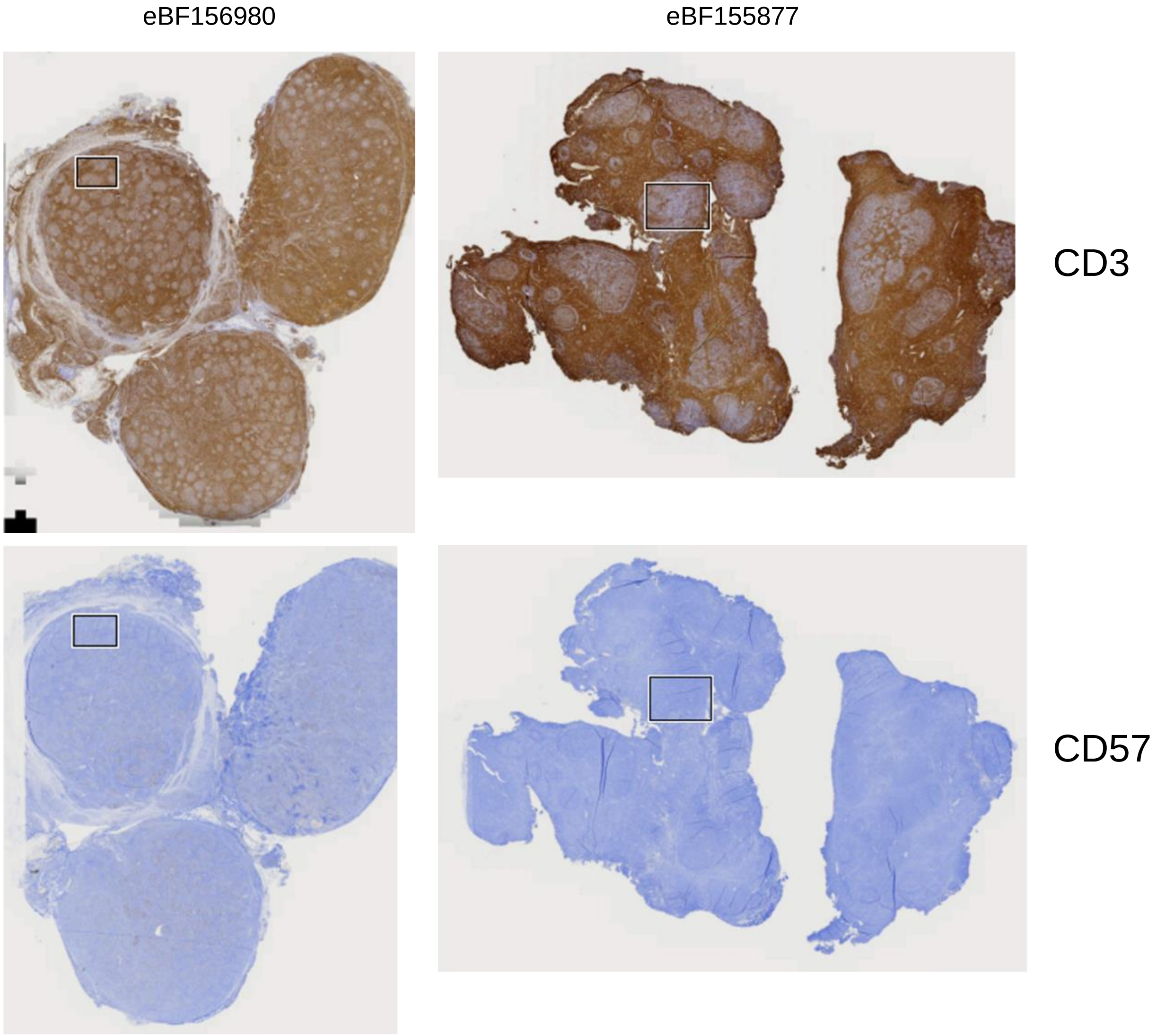

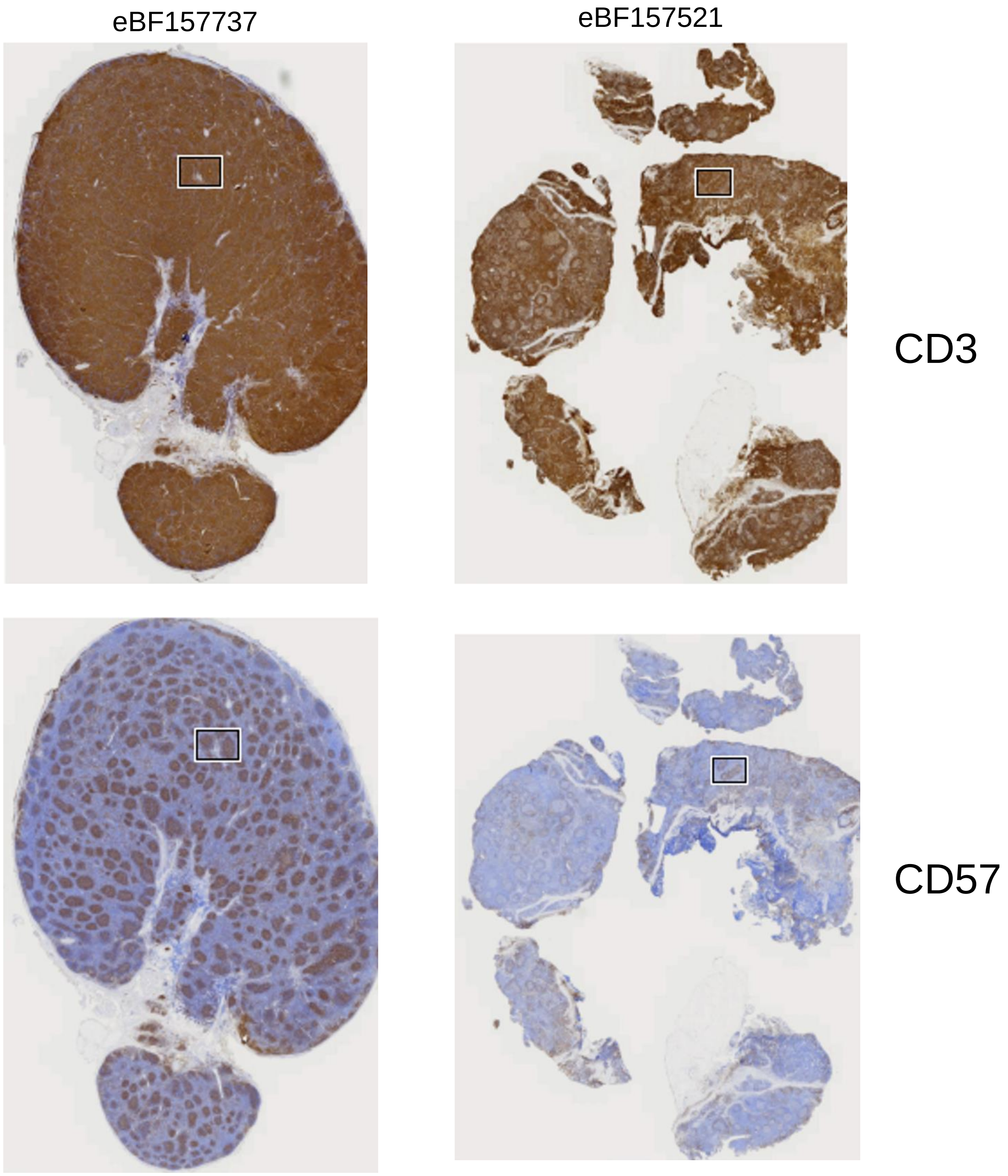
eBF157737
eBF157521
CD3
CD57

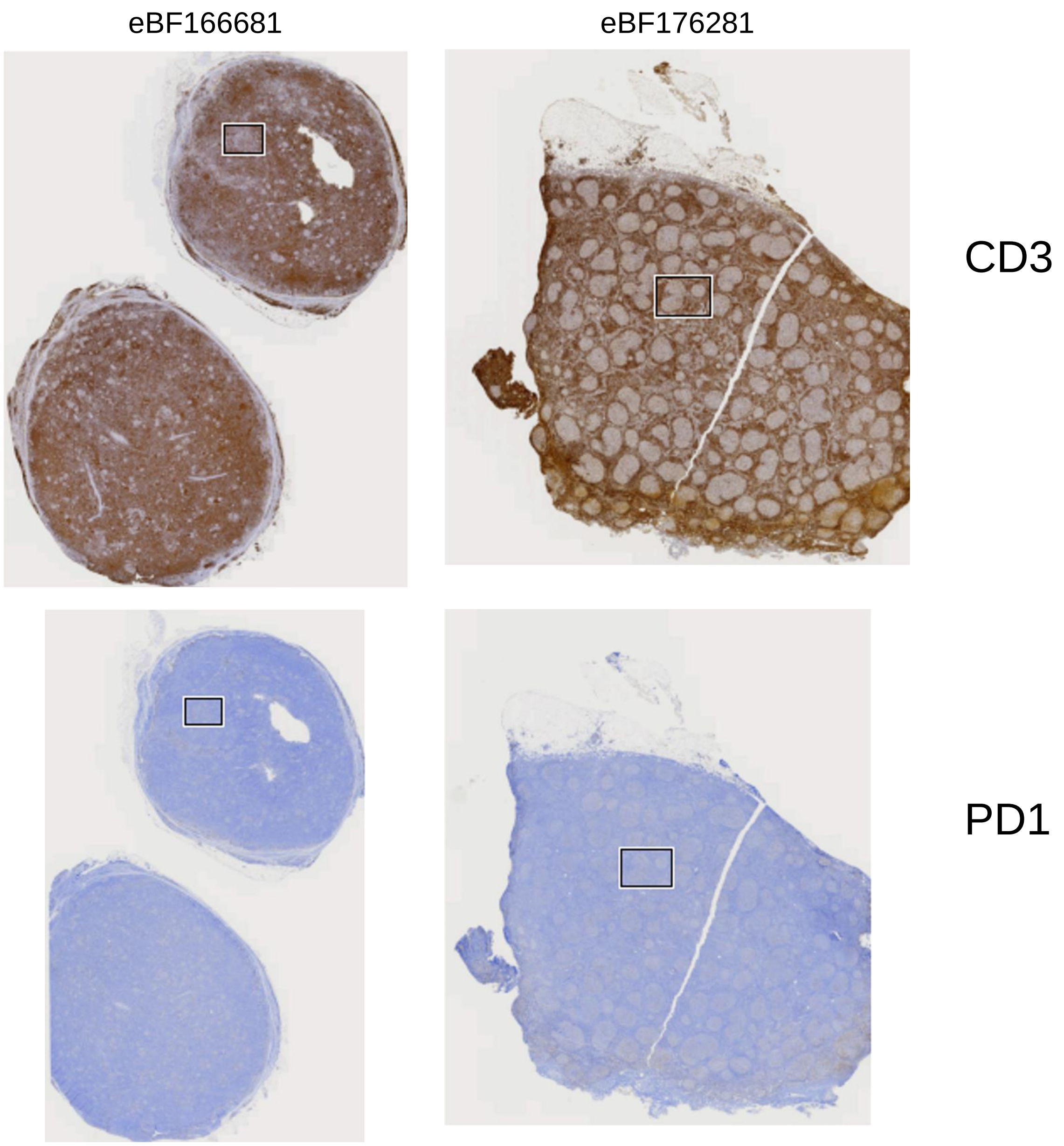
eBF166681
eBF176281
CD3
PD1

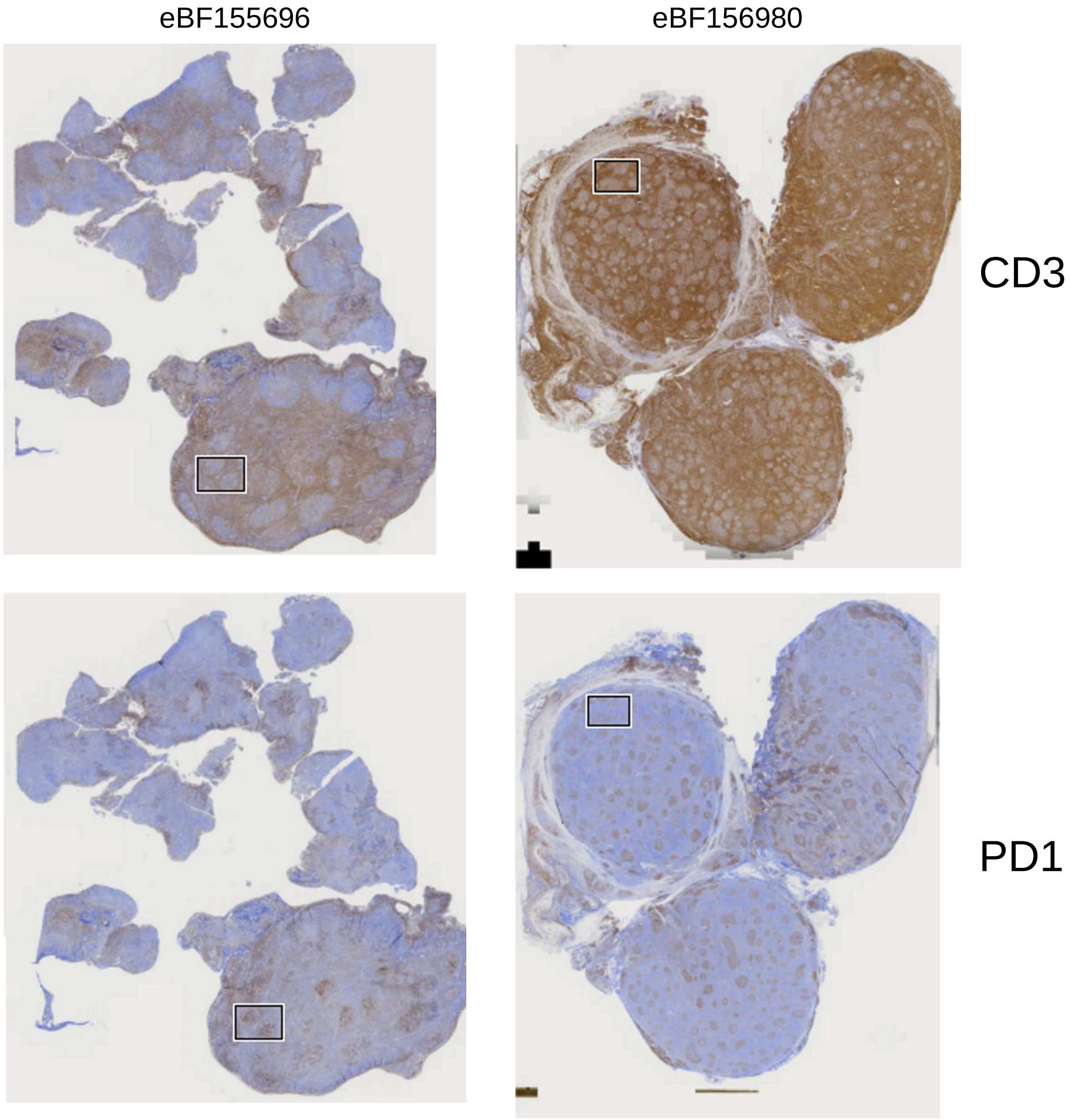
eBF155696
eBF156980
CD3
PD1
