# Supplementary material for: Single-cell profiling reveals a memory B cell-like subtype of follicular lymphoma with increased transformation risk
Source: Nat Commun. 2022 Nov 9;13:6772. doi: 10.1038/s41467-022-34408-0 (PMC9646774; doi:10.1038/s41467-022-34408-0)
Supplement: Supplementary file 3 — Description of Additional Supplementary Files [file 41467_2022_34408_MOESM3_ESM.pdf]

Description of Additional Supplementary Files

Supplementary Data 1. Biopsy site

Supplementary Data 2. FL sample composition by B-cell PG cluster (Raw cell numbers, % of malignant B-cells)

Supplementary Data 3. Intercluster Spearman distances (PG-to-PG level, MC-to-PG level, MC-to-MC level)

Supplementary Data 4. Marker contributions (MC-A vs. MC-B, MC-A vs. not MC-A, MC-B vs. not MC-B, MC-C vs. not MC-C, MC-D vs. not MC-D, MC-E vs. not MC-E, MC-F vs. not MC-F, MC-Mem vs. not MC-Mem, MC-Nav vs. not MC-Nav, MC-Others vs. not MC-Others)

Supplementary Data 5. FL samples (Composition by B-cell MC cluster, Dominant MC cluster)

Supplementary Data 6. Differential gene expression analysis (Gene list from scRNA-seq dataset, Reactome pathways using genes increased in type A from scRNA-seq dataset, Reactome pathways using genes increased in type B from scRNA-seq dataset, Gene list from bulk RNA-seq dataset, Reactome pathways using genes increased in type A from bulk RNA-seq dataset, Reactome pathways using genes increased in type B from bulk RNA-seq dataset)

Supplementary Data 7. Targeted sequencing (Panel composition, Mutation calls, DNA variants, Statistics)

Supplementary Data 8. IGHV somatic hypermutation

Supplementary Data 9. Antibody reagents (CyTOF, Full vs. reduced marker panels, Immunohistochemistry)

Supplementary Data 10. Full network image for Figure 5a.
