# Supplementary figures and images for: Single-cell profiling reveals a memory B cell-like subtype of follicular lymphoma with increased transformation risk
Source: Nat Commun. 2022 Nov 9;13:6772. doi: 10.1038/s41467-022-34408-0 (PMC9646774; doi:10.1038/s41467-022-34408-0)

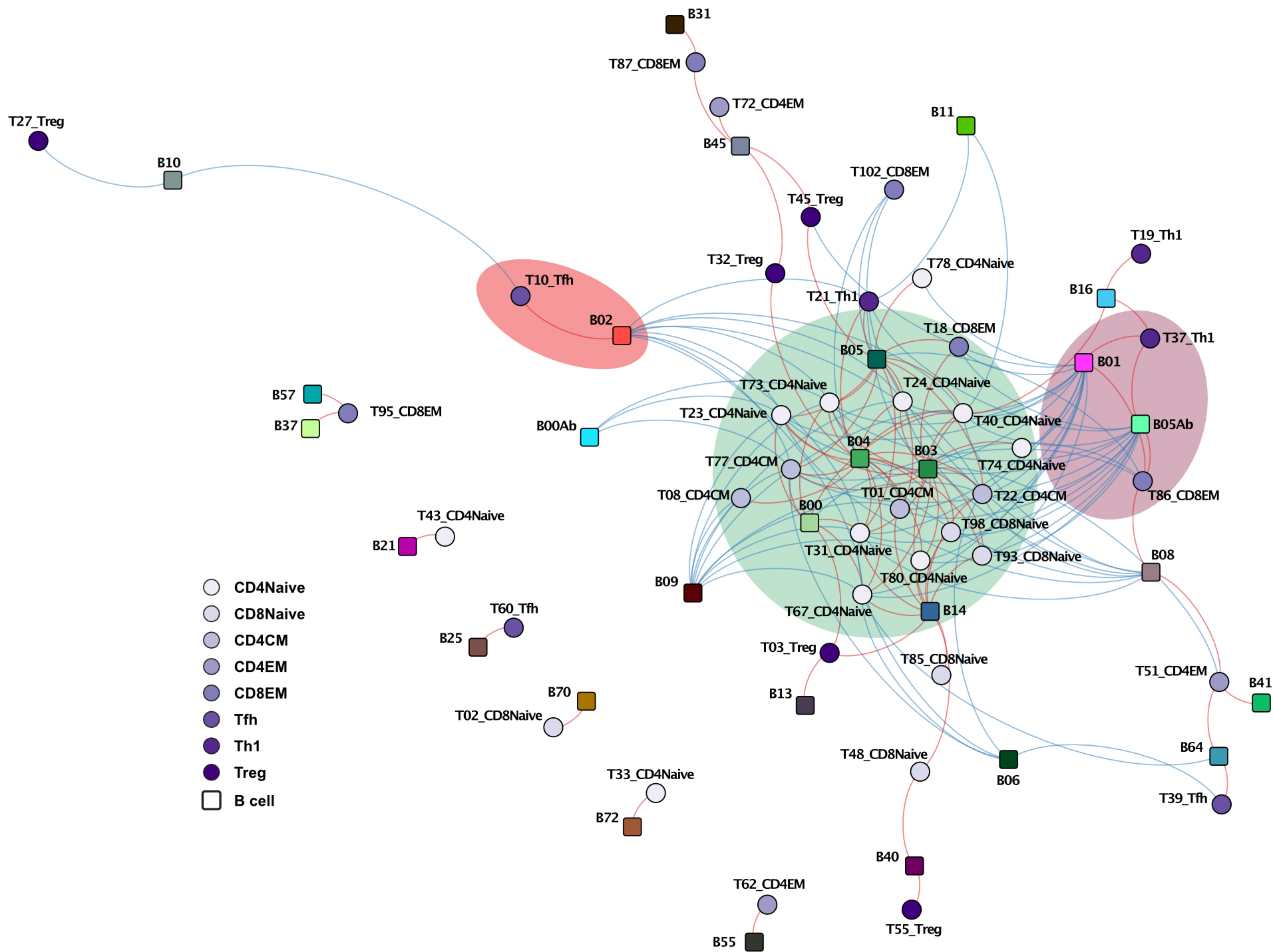

Supplement: Supplementary file 13 — Supplementary Data 10 [file 41467_2022_34408_MOESM13_ESM.pdf]
