# Supplementary material for: Single-cell profiling reveals a memory B cell-like subtype of follicular lymphoma with increased transformation risk
Source: Nat Commun. 2022 Nov 9;13:6772. doi: 10.1038/s41467-022-34408-0 (PMC9646774; doi:10.1038/s41467-022-34408-0)
Supplement: Supplementary file 14 — Reporting Summary [file 41467_2022_34408_MOESM14_ESM.pdf]

nature research

Corresponding author(s): Weng, Andrew

Last updated by author(s): Oct 19, 2022

Reporting Summary

Nature Research wishes to improve the reproducibility of the work that we publish. This form provides structure for consistency and transparency in reporting. For further information on Nature Research policies, see our Editorial Policies and the Editorial Policy Checklist.

Statistics

For all statistical analyses, confirm that the following items are present in the figure legend, table legend, main text, or Methods section.

n/a	Confirmed	
☐	☒	The exact sample size (*n*) for each experimental group/condition, given as a discrete number and unit of measurement
☐	☒	A statement on whether measurements were taken from distinct samples or whether the same sample was measured repeatedly
☐	☒	The statistical test(s) used AND whether they are one- or two-sided<br>*Only common tests should be described solely by name; describe more complex techniques in the Methods section.*
☐	☒	A description of all covariates tested
☐	☒	A description of any assumptions or corrections, such as tests of normality and adjustment for multiple comparisons
☐	☒	A full description of the statistical parameters including central tendency (e.g. means) or other basic estimates (e.g. regression coefficient) AND variation (e.g. standard deviation) or associated estimates of uncertainty (e.g. confidence intervals)
☐	☒	For null hypothesis testing, the test statistic (e.g. *F*, *t*, *r*) with confidence intervals, effect sizes, degrees of freedom and *P* value noted<br>*Give P values as exact values whenever suitable.*
☒	☐	For Bayesian analysis, information on the choice of priors and Markov chain Monte Carlo settings
☒	☐	For hierarchical and complex designs, identification of the appropriate level for tests and full reporting of outcomes
☐	☒	Estimates of effect sizes (e.g. Cohen's *d*, Pearson's *r*), indicating how they were calculated

Our web collection on statistics for biologists contains articles on many of the points above.

Software and code

Policy information about availability of computer code

Data collection	CyTOF software v6.0.626 (Fluidigm)
Data analysis	MATLAB v8.6; Normalizer v0.3; Single-cell Debarcoder (https://github.com/zunderlab/single-cell-debarcoder); FlowJo v10; R v3.3-v3.5 & v4.1.1; Python v3.6; CATALYST v1.10.3; FlowCore v1.52.1; umap-learn v0.3-v0.4; Rtsne v0.15; Phenograph v1.5.2; aricode v1.0.0; iGraph v1.2.6; Gephi v0.9.2; SAM v3.0; cooccur v1.3; ImageScope v12.4.3.5008; CellRanger v2.1.0; Scater v1.8.0; Scran v1.9.11; Scanpy v1.6.0; STAR v2.5.2.a; HTSEQ-count v0.11.0; edgeR v3.22.2; DESeq2 v1.34; limma v3.50.3; pheatmap v1.0.12; BWA v0.7.5a, Mutascope v1.02, SAMtools v0.1.19; Trinity v2.1.1; IgBLAST v1.14.0; Prism v8.

For manuscripts utilizing custom algorithms or software that are central to the research but not yet described in published literature, software must be made available to editors and reviewers. We strongly encourage code deposition in a community repository (e.g. GitHub). See the Nature Research guidelines for submitting code & software for further information.

Data

Policy information about availability of data

All manuscripts must include a data availability statement. This statement should provide the following information, where applicable:

- Accession codes, unique identifiers, or web links for publicly available datasets
- A list of figures that have associated raw data
- A description of any restrictions on data availability

Source CyTOF datafiles are available on FlowRepository under accession #FR-FCM-Z3EL [https://flowrepository.org/id/FR-FCM-Z3EL]. These data are associated with Figures 1, 3, 4, and 5.

scRNA-seq BAM files (generated with CellRanger v2.1.0) for the 4 rLN samples have previously been deposited in the European Genome-phenome Archive (EGA)

under accession #EGAS00001004085 [https://ega-archive.org/studies/EGAS00001004085]67. scRNA-seq BAM files for the 6 FL samples have been deposited into EGA under accession #EGAS00001005257 [https://ega-archive.org/studies/EGAS00001005257]. Access to these data is restricted to qualified investigators due to patient privacy concerns relating to potentially identifiable sequence-level information. Access can be requested from the Data Access Committee via the EGA portal with data made available to qualified investigators within approximately 2 months. These data are associated with Figure 2.

Bulk RNA-seq FASTQ data files have been deposited in the EGA under accession #EGAS00001006646 . This data is part of an ongoing study, and is also available under restricted access. Access can be requested as above.

Genome alignments were performed against the reference human genome assembly GRCh37/hg19 [https://www.ncbi.nlm.nih.gov/data-hub/genome/GCF_000001405.13/]. Exon junction coordinates were referenced from GENCODE release 19 [https://www.gencodegenes.org/human/release_19.html]. Single nucleotide polymorphisms were identified using dbSNP build 137 [https://www.ncbi.nlm.nih.gov/projects/SNP/snp_summary.cgi?view+summary=view+summars&build_id=137].

Field-specific reporting

Please select the one below that is the best fit for your research. If you are not sure, read the appropriate sections before making your selection.

[x] Life sciences   [ ] Behavioural & social sciences   [ ] Ecological, evolutionary & environmental sciences

For a reference copy of the document with all sections, see nature.com/documents/nr-reporting-summary-flat.pdf

Life sciences study design

All studies must disclose on these points even when the disclosure is negative.

Sample size	No sample-size calculation was performed as we included as many samples as were available to us within the study time window. As this was a discovery phase study, sufficiency of the cohort size was determined after the fact by statistical analysis of observed features in the dataset.
Data exclusions	Only 3 samples from the initial cohort of 155 FL were excluded from clinical outcome analyses due to #1) no clonal B cells were identified in the sample, #2) the patient's histology at transformation was classical Hodgkin lymphoma (rather than DLBCL which is the typical histology seen at transformation), and #3) the initial histology indicated a focus of possible early transformation. Exclusions for samples #2 and #3 were pre-established criteria for outcome analysis, while exclusion of sample #1 was implicit in the experimental analysis design.
Replication	Data reproducibility was assessed by two different approaches. First, a spiked-in aliquot from a master pool of control rLN cells was included in each of the CyTOF staining and acquisition runs. Second, a subset of samples were acquired on two separate occasions in different staining/acquisition runs. In both cases, the sample replicates were highly similar to one another indicating a high degree of data reproducibility.
Randomization	Randomization into experimental groups is not a relevant feature of this study since all available samples were assayed and reported on in their entirety.
Blinding	Our study is a retrospective cohort study without blinding in order that clinical outcome associations could be performed.

Reporting for specific materials, systems and methods

We require information from authors about some types of materials, experimental systems and methods used in many studies. Here, indicate whether each material, system or method listed is relevant to your study. If you are not sure if a list item applies to your research, read the appropriate section before selecting a response.

Materials & experimental systems

n/a	Involved in the study	
[ ]	[x]	Antibodies
[x]	[ ]	Eukaryotic cell lines
[x]	[ ]	Palaeontology and archaeology
[x]	[ ]	Animals and other organisms
[ ]	[x]	Human research participants
[ ]	[x]	Clinical data
[x]	[ ]	Dual use research of concern

Methods

n/a	Involved in the study	
[x]	[ ]	ChIP-seq
[x]	[ ]	Flow cytometry
[x]	[ ]	MRI-based neuroimaging

Antibodies

Antibodies used	CD3 (clone UCHT1 & polyclonal; DVS cat#3170001B & Dako cat#GA50361-2), CD4 (clone SK3; DVS cat#3174004B), CD8 (clone HIT8a; BD Biosciences cat#555630), CD10 (clone HI10a; DVS cat#3156001B), CD19 (clone HIB19; DVS cat#3142001B), CD20 (clone 2H7; DVS cat#3147001B), CD21 (clone BL13; DVS cat#3152010B), CD22 (clone HIB22; DVS cat#3159005B), CD23 (clone EBVCS2; eBioscience cat#14-0238-82), CD24 (clone ML5; DVS cat#3166007B), CD25 (IL-2R) (clones 2A3 & MA-251; DVS cat#3169003B & Biolegend cat#356102), CD27 (clones O323 & L128; DVS cat#3167002B & DVS cat#3167006B), CD28 (clone CD28.2; Biolegend cat#302934),

CD30 (clone Ber-H2; eBioscience cat#14-0309-82), CD38 (clone HIT2; DVS cat#3172007B), CD40 (clone 5C3; DVS cat#3165005B), CD43 (clone 84-3C1; DVS cat#3150006B), CD44 (clone IM7; DVS cat#3171003B), CD45 (clone HI30; DVS cat#3089003B), CD45RA (clone HI100; Biolegend cat#304143), CD45RO (clone UCHL1; Biolegend cat#304202), CD48 (clone TÜ145; BD Biosciences cat#555758), CD49D (clone 9F10; DVS cat#3141004B), CD56 (clone NCAM16.2; DVS cat#3149021B), CD57 (clones HCD57 & TB01; Biolegend cat#322302 & Dako cat#GA64761-2), CD69 (clone FN50; DVS cat#3162001B), CD72 (clone 3F3; DVS cat#3144005B), CD79B (clone CB3-1; BD Biosciences cat#555678), CD80 (clone 2D10.4; DVS cat#3161023B), CD83 (clone HB15; Biolegend cat#305302), CD84 (clone CD84.1.21; DVS cat#3154013B), CD86 (clone IT2.2; BD Biosciences cat#555663), CD107a (clone H4A3; DVS cat#3151002B), CD124 (IL-4R) (clone hIL4R-M57; BD Biosciences cat#551894), CD127 (IL-7R) (clone A019D5; DVS cat#3176004B), CD134 (OX40) (clone ACT35; DVS cat#3158012B), CD137 (4-1BB) (clone 4B4-1; Biolegend cat#309802), CD152 (CTLA-4) (clone 14D3; DVS cat#3161004B), CD154 (CD40L) (clone 24-31; DVS cat#3168006B), CD159a (NKG2A) (clone Z199; DVS cat#3169013B), CD160 (clone BY55; Biolegend cat#341202), CD184 (CXCR4) (clone 12G5; DVS cat#3175001B), CD185 (CXCR5) (clone RF8B2; BD Biosciences cat#552032), CD194 (CCR4) (clone 205410; DVS cat#3158006A), CD195 (CCR5) (clone NP-6G4; DVS cat#3156015A), CD197 (CCR7) (clone G043H7; DVS cat#3159003A), CD200 (clone OX-104; DVS cat#3149007B), CD223 (LAG3) (clones 874501 & 17B4; DVS cat#3150016B & Enzo cat#ALX-804-806-C100), CD272 (BTLA) (clone MIH26; DVS cat#3163009B & Biolegend cat#344502), CD274 (PD-L1) (clone 29E.2A3; DVS cat#3148017B), CD278 (ICOS) (clone C398.4A; DVS cat#3148019B), CD279 (PD-1) (clones EH12.2H7 & NAT105; DVS cat#3155009B & Cell Marque cat#315M), CD314 (NKG2D) (clone 1D11; Biolegend cat#320802), CD357 (GITR) (clone 621; Biolegend cat#311602), HLA-ABC (clone W6/32; Biolegend cat#311402), HLA-DR (clone L243; DVS cat#3174001B), HLA-E (clone 3D12; Biolegend cat#342602), IFN-gamma (clone B27; DVS cat#3165002B), Ig kappa (clone MHK-49; DVS cat#3160005B), Ig lambda (clone MHL-38; DVS cat#3151004B), IgD (clone IA6-2; Biolegend cat#348202), IgG (clone G18-145; BD Biosciences cat#555784), IgM (clone MHM-88; Biolegend cat#314527), IL-17A (clone N49-653; DVS cat#3164002B), IL-2 (clone MQ1-17H12; DVS cat#3166002B), IL-21 (clone 3A3-N2; DVS cat#3172011B), IL-4 (clone MP4-25D2; DVS cat#3144010B), IL-6 (clone MQ2-13A5; DVS cat#3147002B), TIGIT (clone MBSA43; DVS cat#3153019B), TIM-3 (clones F38-2E2 & 344823; DVS cat#3154010B & R&D cat#MAB2365), TNF-alpha (clone Mab11; DVS cat#3152002B).

Validation	All primary antibodies were obtained from commercial suppliers who performed validation for human species and cognate antigen specificity in flow/mass cytometry or immunohistochemistry applications and/or provided primary literature references. All primary antibodies were further tested and validated using human reactive lymph node samples or cell lines in mass cytometry or immunohistochemistry applications. Validation experiments were also performed to assess effects of the barcoding reagent on antibody staining by comparing cells stained with antibody before and after the barcoding step. Antibodies that were negatively affected by the barcoding step were used before barcoding on individual samples; samples were then barcoded and pooled, then batch stained with the remaining antibodies in the panel.

Human research participants

Policy information about studies involving human research participants

Population characteristics	Relevant co-variates among the cohort of 155 FL patients are provided in Table S1 and Supplementary Data 1. We obtained genotype information on a subset of patients by targeted panel sequencing of DNA extracted from initial diagnostic/pre-treatment biopsy specimens (Figure 6, Supplementary Data 7).
Recruitment	Patient samples were selected from available cryopreserved single-cell suspension material remaining after diagnostic flow cytometry assessment performed at BCCA. Given that ~3 million live cells per sample were required for CyTOF assessment, our selection of case material was inherently biased towards patients with larger biopsies where sufficient material remained for cryopreservation after completion of diagnostic testing. Comparison of the CyTOF patient cohort to all FL patients seen at our institution over the same time period revealed that the CyTOF cohort was statistically enriched for younger patients with larger tumor masses and who were more likely to have received primary systemic therapy (see Table S1). Further studies are needed to validate our findings in more representative patient cohorts.
Ethics oversight	Informed consent or consent waiver was obtained for all samples utilized for research according to protocols approved by the University of British Columbia/BC Cancer Agency Research Ethics Board.

Note that full information on the approval of the study protocol must also be provided in the manuscript.

Clinical data

Policy information about clinical studies

All manuscripts should comply with the ICMJEguidelines for publication of clinical research and a completedCONSORT checklist must be included with all submissions.

Clinical trial registration	This was not a clinical trial.
Study protocol	For this retrospective study, inclusion criteria were defined as stated in the manuscript and in this form. Briefly, patients were required to have received a tissue diagnosis of FL, and for fresh frozen cell suspension material from the diagnostic FL biopsy to have been banked prior initiation of therapy.
Data collection	Fresh frozen cell suspension material was obtained from the BCCA Lymphoid Cancer tissue bank between 2013 and 2017. Clinical and demographic data were obtained from the BCCA Lymphoid Cancer clinical and pathology databases between 2013 and 2020.
Outcomes	We estimated the time to progression (TTP; progression/relapse or death from lymphoma or acute treatment toxicity), time to transformation (TTT; biopsy-proven histologic or clinically determined transformation), disease-specific survival (DSS; death from lymphoma or acute treatment toxicity) and overall survival (OS; death from any cause) for outcome analyses.
